# Endogenous Abscisic Acid Promotes Hypocotyl Growth and Affects Endoreduplication during Dark-Induced Growth in Tomato (*Solanum lycopersicum* L.)

**DOI:** 10.1371/journal.pone.0117793

**Published:** 2015-02-19

**Authors:** Jan F. Humplík, Véronique Bergougnoux, Michaela Jandová, Jan Šimura, Aleš Pěnčík, Ondřej Tomanec, Jakub Rolčík, Ondřej Novák, Martin Fellner

**Affiliations:** 1 Laboratory of Growth Regulators & Department of Chemical Biology and Genetics, Centre of the Region Haná for Biotechnological and Agricultural Research, Faculty of Science, Palacký University & Institute of Experimental Botany ASCR, Olomouc, Czech Republic; 2 Department of Molecular Biology, Centre of the Region Haná for Biotechnological and Agricultural Research, Faculty of Science, Palacký University, Olomouc, Czech Republic; 3 Department of Botany, Faculty of Science, Palacký University, Olomouc, Czech Republic; 4 Regional Centre of Advanced Technologies and Materials, Department of Physical Chemistry, Palacký University, Olomouc, Czech Republic; Estación Experimental del Zaidín (CSIC), SPAIN

## Abstract

Dark-induced growth (skotomorphogenesis) is primarily characterized by rapid elongation of the hypocotyl. We have studied the role of abscisic acid (ABA) during the development of young tomato (*Solanum lycopersicum* L.) seedlings. We observed that ABA deficiency caused a reduction in hypocotyl growth at the level of cell elongation and that the growth in ABA-deficient plants could be improved by treatment with exogenous ABA, through which the plants show a concentration dependent response. In addition, ABA accumulated in dark-grown tomato seedlings that grew rapidly, whereas seedlings grown under blue light exhibited low growth rates and accumulated less ABA. We demonstrated that ABA promotes DNA endoreduplication by enhancing the expression of the genes encoding inhibitors of cyclin-dependent kinases *SlKRP1* and *SlKRP3* and by reducing cytokinin levels. These data were supported by the expression analysis of the genes which encode enzymes involved in ABA and CK metabolism. Our results show that ABA is essential for the process of hypocotyl elongation and that appropriate control of the endogenous level of ABA is required in order to drive the growth of etiolated seedlings.

## Introduction

**Fig 1:**
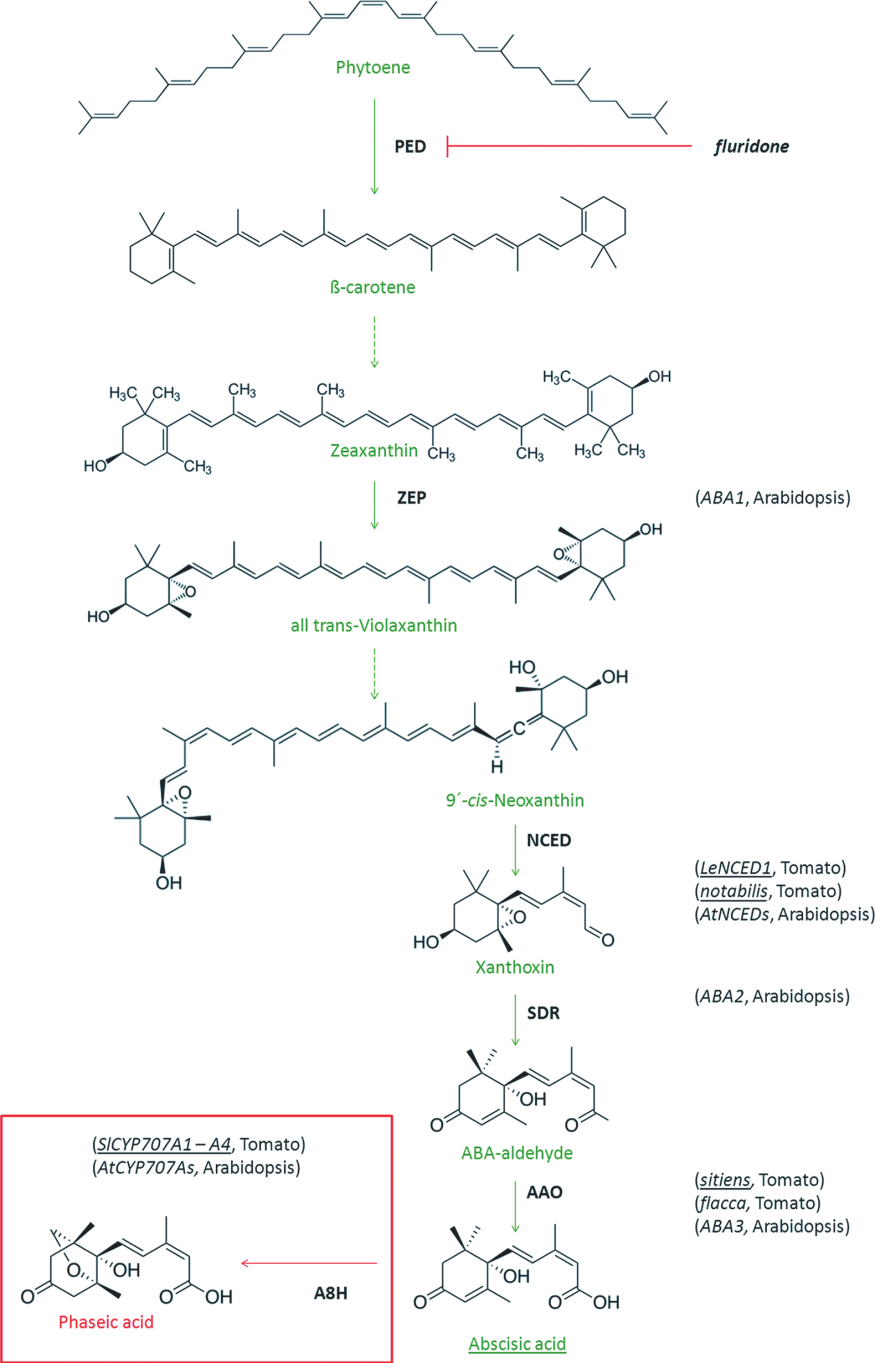
Simplified scheme of ABA biosynthesis and catabolism. Selected enzymatic steps in ABA biosynthesis are shown. The names of the genes encoding the enzymes that catalyze each step in tomato and *Arabidopsis* are indicated; the names of genes examined in this work are underlined. The conversion of phytoene to ß-carotene is mediated by phytoene desaturase (PED); this step is blocked by fluridone. Zeaxanthin epoxidase (ZEP) catalyzes the synthesis of violaxanthin, which is then converted to neoxanthin. The subsequent synthesis of xanthoxin is catalyzed by 9-*cis*-epoxycarotenoid dioxygenase (NCED), which is encoded in the gene *LeNCED1* in tomato and disrupted in *notabilis* mutant. Whereas the previous steps occur in plastids, xanthoxin is transported to the cytosol where it is converted to the abscisic aldehyde by short-chain dehydrogenase/reductase (SDR). The final step of ABA biosynthesis is the oxidation of abscisic aldehyde to ABA by an abscisic aldehyde oxidase (AAO), which is encoded in genes that are disrupted in the *sitiens* and *flacca* tomato mutants. ABA degradation (shown in the red frame) is mediated by ABA 8´-hydroxylase (A8H, cytochrome P450 monooxygenase), whose product spontaneously isomerizes to phaseic acid. The genes encoding ABA 8´-hydroxylase in tomato are *SlCYP707A1–SlCYP707A4*. Dashed arrows represent missing steps in the pathway. These schemes are modified according to Kitahata and co-authors [32] and Nambara and Marion-Poll [70].

Abscisic acid (ABA) is very often regarded as an inhibitor of shoot growth e. g. [1], [2], [3]. This is based on the fact that i) ABA accumulates at high concentrations in water stressed plants, correlating with growth inhibition [4], [5], [6] and ii) treatment with exogenous ABA at μM concentrations inhibits shoot growth [7], [5], [8]. However, ABA deficient mutants are shorter than the corresponding wild-type (WT) plants, and their growth can be improved by treatment with exogenous ABA. Their reduced growth was attributed to an impaired water balance [9]. The first evidence that ABA could stimulate shoot growth was obtained in a study on etiolated rice seedlings, in which treatment with extremely low concentrations of exogenous ABA stimulated mesocotyl elongation [10]. Later, Saab and co-authors demonstrated that under conditions of high water potential, the ABA-deficient *viviparous* maize mutant exhibited reduced growth compared to WT plants [11]. Similarly, the ABA biosynthesis-impaired *flacca* tomato mutant exhibited reduced shoot growth and elevated ethylene production compared to the WT. The treatment of the *flacca* mutant with exogenous ABA suppressed its excessive ethylene biosynthesis and restored shoot growth to near WT-levels [12]. The inhibition of vegetative growth was also observed in the *Arabidopsis aba1* and *aba2-1* mutants [13], [14], which are defective in different steps of ABA biosynthesis (Fig. 1). It therefore appears that ABA maintains shoot growth rather than inhibiting it, partly by suppressing ethylene synthesis and partly by some ethylene-independent mechanism.

We focused our study on the role of ABA during tomato seedling development, particularly on the growth of hypocotyl. After a seed germinates in the soil, in the absence of light, the seedling undergoes etiolated growth, known as skotomorphogenesis, which is characterized by rapid elongation of the hypocotyl topped by a hook with underdeveloped cotyledons. When the etiolated seedling perceives the light, it begins photomorphogenesis. The transition from skotomorphogenesis to photomorphogenesis, referred to as de-etiolation, involves several developmental changes such as inhibition of the fast hypocotyl growth, activation of the apical meristem, and the development of the cotyledons and plastids [15]. The role of ABA in skotomorphogenesis is poorly documented. Barrero et al. (2008) reported that dark-grown *Arabidopsis aba1* seedlings, deficient in ABA-biosynthesis, had a de-etiolated phenotype [16]. However, since this mutant is also impaired in carotenoid synthesis, the authors concluded that one of ABA’s carotenoid biosynthetic precursors was responsible for this effect rather than the ABA itself.

In this work, we investigated the role of ABA during skotomorphogenesis in tomato seedlings (*Solanum lycopersicum* L). Our study was intended to answer the question: Does ABA contribute to the rapid stem growth observed during skotomorphogenesis or does it play a role in growth inhibition observed during tomato de-etiolation? Using physiological and genetic approaches we demonstrated that finely-tuned regulation of ABA homeostasis is required to promote or inhibit growth. Indeed, ABA was found to promote hypocotyl elongation of etiolated ABA deficient tomato seedlings that exhibited a concentration-dependent response. The results were also supported by the analysis of ABA content, and the expression of ABA metabolic genes in contrasting developmental situations. It seems that ABA stimulates cell expansion by enhancing endoreduplication via the elevated expression of cyclin-dependent kinases (CDK) inhibitors and the inhibition of cytokinin biosynthesis.

## Materials and Methods

### Plant material and growth conditions

The experiments involving ABA quantification, the analysis of the expression of genes involved in ABA metabolism and pharmacological experiments were performed using wild-type tomato (*Solanum lycopersicum* L.) seedlings of the Rutgers cultivar. In all other experiments that focused on the effects of endogenous ABA deficiency, seedlings of the tomato mutants *sitiens* (*sit*) and *notabilis* (*not*) and the corresponding WTs (cv. Rheinlands Ruhm and cv. Lukullus, respectively) were used. The *sit* mutant is defective in the very last step of ABA biosynthesis [17] (Fig. 1) and consequently produces dramatically less ABA than the corresponding WT [18], [19]. The *not* mutant is impaired in the key regulatory step of ABA biosynthesis, the oxidative cleavage of 9´-*cis*-neoxanthin to xanthoxin [20], and ABA production is also consequently reduced [19], [21].

The seeds were soaked in 3% sodium hypochlorite (Bochemie, Czech Republic) for 20 minutes and rinsed extensively with sterile distilled water prior to sowing. The seeds were then sown on the basal Murashige and Skoog medium [22] supplemented with 0.7% (w/v) agar in square Petri dishes (120 x 120 mm). The pH was adjusted to 6.1 with 1.0 M KOH before autoclaving. The Petri dishes were placed vertically in the dark for 3 days at 23°C to induce germination. For experiments involving blue-light (BL) illumination, the Petri dishes were transferred to a growth chamber (Snijders, The Netherlands) at 23°C with continuous BL illumination provided by fluorescent tubes (TL-D 36W/18-Blue, Philips; total photon ﬂuence rate 10 μmol m^-2^ s^-1^). The light spectrum was measured using a portable spectroradiometer (model LI-1800; Li-COR, NE, USA). For dark conditions, Petri dishes were wrapped in aluminum foil, and placed in the same growth chamber under the same temperature regime.

### Hypocotyl growth measurement

Germinated seeds were transferred to new media that were supplemented as appropriate with abscisic acid or fluridone (both from Sigma-Aldrich, MO, USA) and to new media that were not supplemented with the effectors (control samples). These compounds were added to the medium as 10 mM stock solutions. The stock solution of fluridone was prepared in 10% (v/v) ethanol and the same quantity of solvent was added to the control samples. All samples were placed in a growth chamber for 4 days (96 hours). Hypocotyl length was measured with a ruler and at least 10 seedlings per treatment were measured in each independent experiment. The data of hypocotyl lengths were normalized to the appropriate control samples: the median length of the untreated WT or mutant samples (specified in figure legends) was set as 100% hypocotyl length and all other values were expressed as percentages of this control. The original measured values that were used for data normalization are given in S1 Table. The data are presented as medians and first and third quartiles as they corresponded to the non-parametric statistics. The number of experimental repeats is shown in the figure legends, along with the total number of measured seedlings.

### Epidermal cell length measurement

Seedlings of *sit* and WT (cv. Rheinlands Ruhm) were grown in dark on the basal Murashige and Skoog medium for 4 days after transfer as described above. Then a 1 cm segment from the hypocotyl base was excised and immediately fixed in a solution of 4% formaldehyde, 0.5% glutaraldehyde in a 1x MTSB buffer (50 mM PIPES, 5 mM MgSO4, 5 mM EGTA, pH 6.9). Samples were fixed overnight in a vacuum chamber to enhance the penetration of the fixative solution into the hypocotyl tissue. Subsequently samples were washed twice with the 1x MTSB buffer and dehydrated by increasing the concentration of ethanol solution (15%, 30%, 50%, 75%, 90%, 100%). Each dehydration step lasted 20 minutes. Then the 100% ethanol washing was repeated once and samples were incubated overnight. The solution was then replaced by fresh 100% ethanol solution for storage. Before the analysis hypocotyl segments were mounted on a metal sample holder and ethanol traces evaporated in 3 minutes. Afterwards the sample was immediately transferred to a scanning electron microscope (SEM) and photographed at magnification 100 along the whole length of hypocotyl segment. The epidermal cells were measured in ImageJ software [23] and a non-parametric variant of the t-test (Mann-Whitney test) was applied to prove the significance of the obtained data. The data are presented as medians and first and third quartiles. For each genotype at least 230 cells from three representative hypocotyls were analyzed.

### Extraction and quantification of ABA

The seeds and seedlings were cultivated in the dark as described above. For the analysis of ABA, seedlings were harvested at 6, 12, 24, 48, 72, 96 and 120 hours after sowing. To obtain de-etiolated seedlings for ABA analysis, the Petri dishes were transferred to BL after seed germination, which occurred 72 hours after sowing. Prior to germination at 72 hours, all seeds were collected; after 72 hours, only germinated seeds were used for analysis. For the analysis in the ABA-deficient mutants, the hypocotyls were excised and frozen immediately in liquid nitrogen. The dark-grown samples were harvested under safety green light while BL-grown samples were harvested under BL illumination. The collected samples were homogenized with a mortar and pestle in liquid nitrogen. For determination of the endogenous levels of ABA, 10 mg of homogenized tissue were extracted by using a phosphate buffer. The sample was then purified by solid-phase extraction on a C8 column as described previously for auxins [24], except in this case [^2^H_6_]ABA was added as an internal standard. After evaporation under reduced pressure, samples were analyzed for free ABA content by UPLC (Acquity UPLC^TM^; Waters, USA) linked to a triple quadrupole mass detector (Xevo TQ MS^TM^; Waters, USA). The same trend in relative ABA content was observed in all biological repeats; however, the number of repeats was not sufficient for statistical analysis. All data are presented as the mean of the relative ABA content, with the value observed for dark-grown samples at the last time point (120 hours) set as 100 arbitrary units (a.u.) for each experimental repeat. The absolute values obtained in the independent repeats are reported in tables S2 Table and S3 Table.

### Extraction and quantification of CKs

For the quantification of CKs, the etiolated seedlings of *sit* and WT were harvested 4 days after their germination. The collected samples were homogenized with a mortar and pestle in liquid nitrogen. Fifty mg of the sample were then extracted in a modified Bieleski buffer (methanol/ water/formic acid, 15/4/1, v/v/v) [25]. For the purification of free CKs, two SPE columns were used: a C18 octadecylsilica-based column (500 mg of sorbent, Applied Separations) and an MCX column (30 mg of C18/SCX combined sorbent with cation-exchange properties, Waters) [26]. Analytes were eluted by two-step elution using a 0.35 M aqueous solution of NH_4_OH and 0.35 M NH_4_OH in 60% (v/v) MeOH. Samples were then evaporated to dryness under reduced pressure at 37°C. An Acquity UPLC System (Waters, Milford, MA,USA), linked to a triple quadrupole mass spectrometer Xevo^TM^ TQ MS (Waters MS Technologies, Manchester, UK) equipped with an electrospray interface was used to determine cytokinin levels. Stable isotope-labeled CK internal standards (0.5 pmol each for CK bases, ribosides, and *N*-glucosides; 1 pmol each for *O*-glucosides and nucleotides) were added to each sample during the extraction step to enable accurate quantification. The final concentration of each analyte was calculated from its peak area in multiple reaction monitoring mode chromatograms, as described by Svačinová and co-authors [27]. The data are presented as mean relative CK contents, with the measured values for the WT control samples being set to 100 arbitrary units (a.u.) for each experimental repeat. Absolute values for three independent repeats are given in S4 Table.

### Analysis of gene expression by RealTime-qPCR

Seeds and seedlings were cultivated and harvested as described for ABA quantification. Total RNA was extracted using an RNeasy Plant Mini Kit (Qiagen, The Netherlands), with an additional round of DNaseI treatment (Takara, Japan) for 30 minutes at 37°C. The DNase I enzyme was then heat-inactivated at 65°C for 10 minutes, after which the samples were subjected to phenol:chloroform:isoamyl alcohol (25:24:1) purification. The cDNA synthesis was performed using 0.7 μg total RNA with the PrimeScript^TM^ 1^st^ strand cDNA Synthesis Kit (Takara, Japan) according to the manufacturer’s instructions. The RNA was then digested with 5 units of RNaseH (Takara, Japan) for 20 minutes at 37°C. The cDNAs were purified on a column and eluted with RNase/DNase-free distilled water. qPCR reactions were performed using the SYBR Premix Ex Taq kit (Takara, Japan) and 200 nM of each primer. Three technical repeats were performed for each sample in a two-step temperature program. The initial denaturation at 95°C for 10 seconds was followed by 45 cycles of 95°C for 5 seconds and 60°C for 20 seconds. The dissociation curve for each sample was monitored during this time. All Ct values were normalized against those for the *PP2Acs* and *Tip41like* genes [28]. The differences in the cycle numbers of the samples during the linear amplification phase, along with the ΔΔC_T_ method, were used to determine fold changes in gene expression. All results are expressed in term of “fold change”. Relative expression was evaluated using geometrical means calculated from two reference genes in each independent experiment. The quoted values represent the mean relative expressions observed in three independent experiments. While the number of repeats was not sufficient to prove significance in some samples, we observed the same trends in relative gene expression in all experiments (Tables A-D in S1 File). The primer sequences and gene accession numbers in the SOL genomics network (http://solgenomics.net/) are given in Table 1.

**Table 1:** Sequences of primer combinations used in this study.

**Gene name**	**Accession no.**	**Sequence 5´- 3´**	**Reference**
*LeNCED1*	SGN-U577478	F: CTTATTTGGCTATCGCTGAACC	[33]
		R: CCTCCAACTTCAAACTCATTGC	
*SlCYP707A2*	SGN-U583027	F: GCAATGAAAGCGAGGAAAGAG	
		R: TTGTTCGTCAGTGAGTCCTTC	
*SlCYP707A3*	SGN-U585745	F: GCTCCCAAACCCAATACCTAC	[33]
		R: CAGTTTGGCGAGTTCATTTCC	
*SlCYP707A4*	SGN-U583028	F: GCTAGTGTCCTTACATGGATCC	[33]
		R: CTCTCATTATCCCCTCTTGCTC	
*SlKRP1*	AJ441249	F: CAACATTCAGACCCCTGGTT	
		R: CTCCTTTTCTGCACGGGTAA	
*SlKRP3*	SGN-U320533	F: TTCGTACAAGAGCTAAAACCCTAG	
		R: TCTTTTCCCTTCAAACCCCAC	
*SlLOG2*		F: TGTTGGAGAAGTAAGAGCAGTG	[35]
		R: ATGAATGCCTAGTTGAGCCC	
*PP2Acs*	SGN-U567355	F: CGATGTGTGATCTCCTATGGTC	[28]
		R: AAGCTGATGGGCTCTAGAAATC	
*Tip41like*	SGN-U584254	F: GGTTCCTATTGCTGCGTT	[28]
		R: CGAAGACAAGGCCTGAAA	

### Analysis of ploidy—endoreduplication

The seedlings of *sit* mutant and WT were grown in the dark as described above. The hypocotyls were harvested and immediately used to determine the level of DNA endoreduplication using a C6 Flow cytometer (Accuri BD, New Jersey, USA) with a blue laser (488 nm). Ten mm-long segments were excised from the upper part of the hypocotyl and cut with a razor blade in a Petri dish containing 550 μl of ice cold LB01 buffer [29]. The suspension of nuclei was then filtered through a 42 μm nylon mesh and stained with propidium iodide (50 μg.ml^−1^). For each sample, 5000 particles were measured. A histogram of fluorescence intensity was registered on the FL2 channel using a linear scale. Because the first peak in the histogram corresponded to the G1 (2C) phase of the cell cycle, the ploidy levels of the other peaks were determined by comparison with the position of the G1 peak. The average ratio of the G1 and G2 peak positions was 1.97 (4C) and that of the G1 and G3 positions was 3.69 (8C) with coefficients of variance ranging from 3.7–9.0. For each of the four studied treatments, a total of 10 seedlings were analyzed in three independent experiments.

### Statistical analysis

When comparing the results for two samples, the non-parametric variant of the t-test (Mann-Whitney test) was used to assess statistical significance. For multiple-sample experiments, significance was assessed using the non-parametric variant of one-way ANOVA, Kruskal-Wallis test with multiple comparisons post-hoc. The obtained differences were considered as significant when the *p*-value was lower than 0.05 (95% reliability), but in many cases the *p*-values were lower than 0.01 (99% reliability), particular *p*-values are given in figure legends. All statistical analyses were performed using the STATISTICA 12 software (StatSoft, OK, USA). When the number of repeats was not sufficient to prove significance (gene expression and hormone analysis), the original values used for calculation of the means and SE are given in the Supporting information to demonstrate that the data of all replicates in particular experiment showed the same trends.

## Results

### The effects of ABA deficiency on hypocotyl growth and expansion of hypocotyl cells

**Fig 2:**
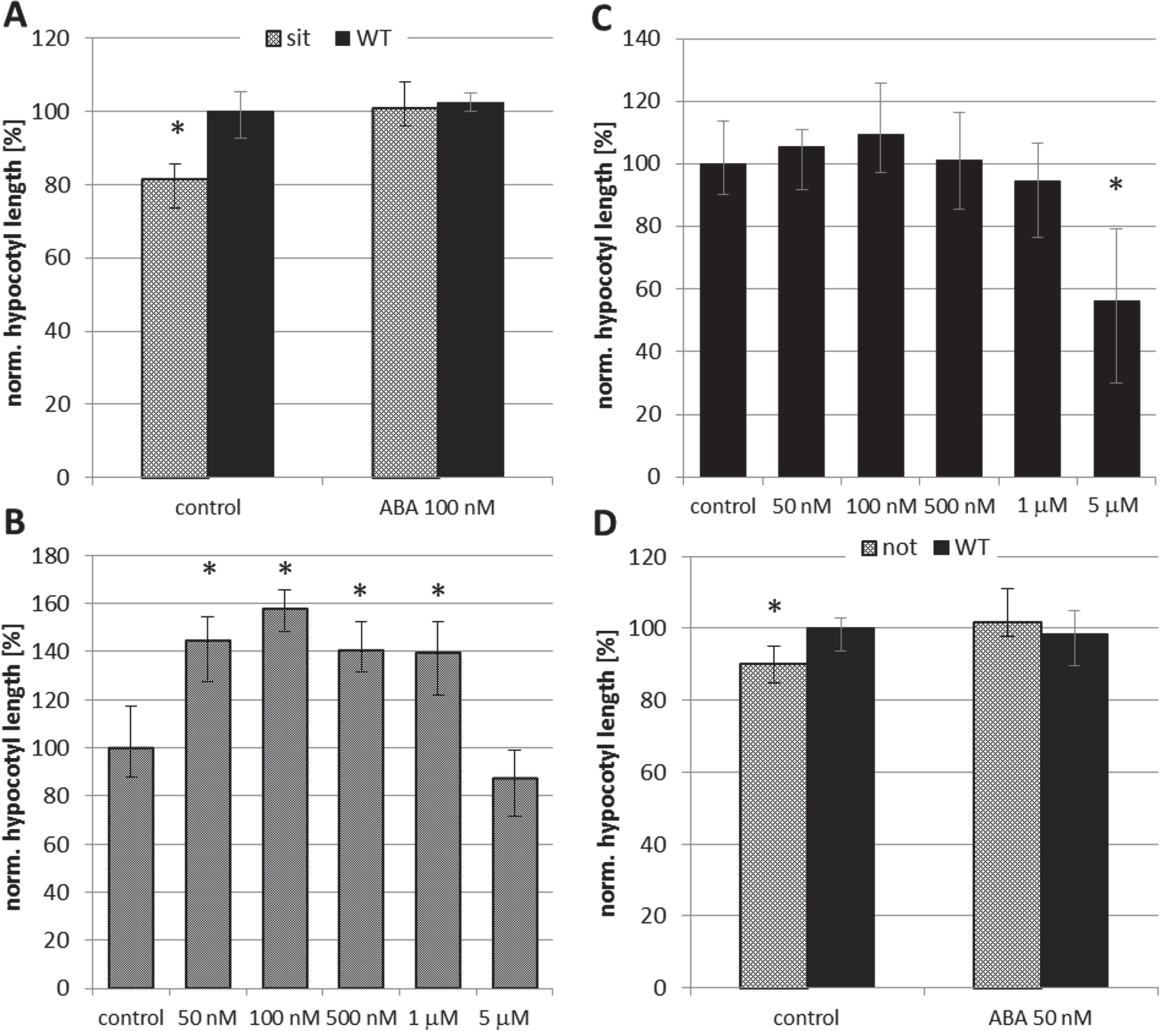
The effects of ABA on the growth of etiolated hypocotyls. A) The effect of ABA-deficiency and exogenous ABA on hypocotyl growth in the *sit* mutant. Germinated seeds of the WT (cv. Rheinlands Ruhm) mutant were transferred to untreated media (control) and media supplemented with 100 nM ABA and grown in the dark for 4 days. The results shown in the figure represent the medians of normalized length of hypocotyls from three independent experiments; the error bars represent the boundaries of the first and third quartiles. The “WT control” was set as 100% hypocotyl length and all other values (medians, quartiles) are expressed as percentages of this value. To prove statistical significance the Mann-Whitney test was performed independently (*sit* versus WT) for control and ABA treated samples. Asterisks denote values that differ significantly (Mann Whitney test; p < 0.01, n = 136). B) The hypocotyl elongation of the *sit* mutant treated with various concentrations of ABA. Germinated seeds were transferred to untreated media (control) and media supplemented with ABA and grown in the dark for 4 days. The results shown in the figure represent the medians of normalized length of hypocotyls from two independent experiments; the error bars represent the boundaries of the first and third quartiles. The sample “control” was set as 100% hypocotyl length and all other values (medians, quartiles) are expressed as percentages of this value. To prove statistical significance the Kruskal-Wallis ANOVA with multiple post-hoc comparison was performed. Asterisks denote values that differ significantly from “control” sample (p < 0.01; n = 232). C) The hypocotyl elongation of the WT (cv. Rheinlands Ruhm) treated with various concentrations of ABA. Germinated seeds were transferred to untreated media (control) and media supplemented with ABA and grown in the dark for 4 days. The results shown in the figure represent the medians of normalized length of hypocotyls from two independent experiments; the error bars represent the boundaries of the first and third quartiles. The sample “control” was set as 100% hypocotyl length and all other values (medians, quartiles) are expressed as percentages of this value. To prove statistical significance the Kruskal-Wallis ANOVA was performed. Asterisks denote values that differ significantly from “control” sample (p < 0.01, n = 200). D) The effect of ABA-deficiency and exogenous ABA on hypocotyl growth in the *not* mutant. Germinated seeds of the WT (cv. Lukullus) mutant were transferred to untreated media (control) and media supplemented with 50 nM ABA and grown in the dark for 4 days. The results shown in the figure represent the medians of normalized length of hypocotyls from one independent experiment. The “WT control” was set as 100% hypocotyl length and all other values (medians, quartiles) are expressed as percentages of this value. To prove statistical significance the Mann-Whitney test was performed independently (*not* versus WT) for control and ABA treated samples. Asterisks denote values that differ significantly (Mann Whitney test; p < 0.03, n = 79).

**Fig 3:**
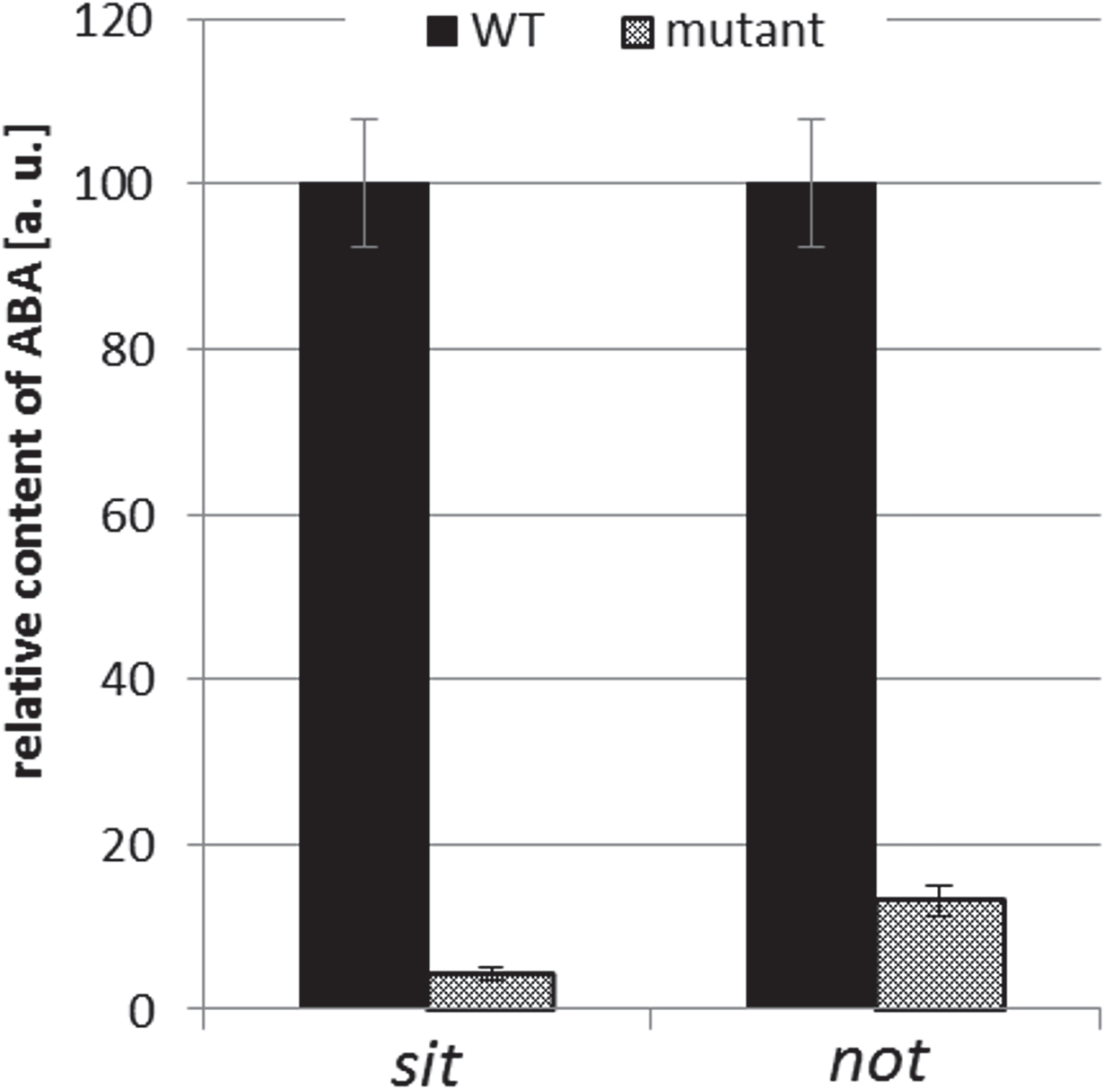
Effect of *sit* and *not* mutations on the endogenous ABA content in etiolated hypocotyls. The dark-grown hypocotyls of *sit* and *not* mutants and corresponding WTs were harvested five days after germination and ABA content was measured. Free ABA levels are reported as relative means [a.u.] ± SE based on two biological repeats. The mutant values are expressed relative to those for corresponding WT, which were assigned a value of 100 a.u.

The tomato *sit* mutant, which is defective in the last step of ABA biosynthesis, was used as the main tool to investigate the role of ABA in hypocotyl growth. The dark-grown *sit* mutant produced significantly shorter hypocotyls than WT plants grown under identical conditions (Fig. 2A). However, when ABA was added to the growth medium at a concentration of 100 nM, the hypocotyls of the *sit* mutant grew to the same length as those of WT plants. When different concentrations of ABA (50 nM to 5 μM) were applied the hypocotyl length was significantly stimulated by nM but inhibited by concentrations higher than 1μM in the *sit* mutant (Fig. 2B). On the other hand in the WT inhibition only was observed at the highest ABA concentration treatment (Fig. 2C). A similar response was observed in *not* mutant and corresponding WT (cv. Lukullus). The *not* seedlings were significantly inhibited in the hypocotyl growth compared to the WT, but the treatment with 50 nM ABA led to the improvement of the mutant. WT seedlings did not respond to the exogenous ABA treatment (Fig. 2D). In comparison with *sit* mutant, a lower ABA concentration (50 nM) was chosen as most efficient for *not*, based on the previous test of ABA sensitivity (S1 Fig.). ABA contents in *sit* and *not* mutant and corresponding WT hypocotyls were analyzed to see the effects of mutations in our experimental conditions. Both mutants showed dramatically lowered endogenous ABA compared to the WT samples (Fig. 3, S3 Table). In order to determine the role of ABA during photomorphogenesis, additional experiments were conducted using seedlings grown under BL illumination, which is known to strongly inhibit hypocotyl growth [30], [31]. The *sit* mutation did not have any discernible effect on hypocotyl growth under continuous BL illumination (S2 Fig.).

**Fig 4:**
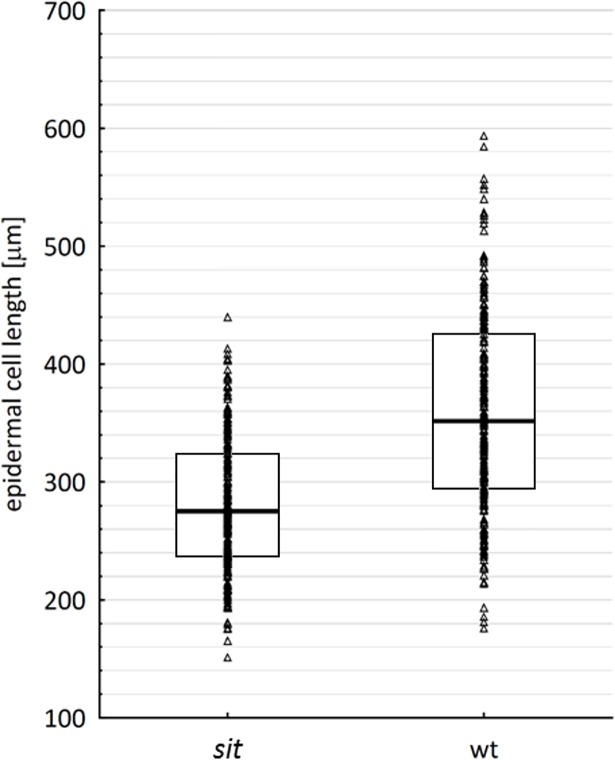
Effect of ABA deficiency on the expansion of hypocotyl cells. The influence of ABA on the elongation of epidermal cells of etiolated hypocotyls in *sit* and WT (cv. Rheinlands Ruhm) was investigated using SEM imaging analysis. Triangles represent all measured values (n = 471) for each genotype. The bars within the boxes indicate the median values in each case, while the boxes’ upper and lower boundaries indicate the boundaries of the first and third quartiles. The Mann-Whitney test was used to prove statistical significance (p < 0.01).

In parallel to the studies using mutant lines, we conducted a series of experiments using synthetic inhibitor of ABA biosynthesis. Freshly germinated tomato (cv. Rutgers) seedlings were grown in the dark for four days on a medium supplemented with fluridone (10 μM) and then the length of the hypocotyls was measured. Fluridone is an inhibitor of carotenoid pathway, suppressing early steps of ABA synthesis [32] (for details see Fig. 1). The analysis of ABA content in dark-grown WT hypocotyls showed that fluridone lowered endogenous ABA to the less than 50% of the control samples (S3 Fig.). Fluridone caused significant inhibition of hypocotyl growth in the etiolated seedlings (S4 Fig.). To see how ABA deficiency affected hypocotyl cell elongation the microscopic experiments were performed. The measurement of cell length showed significant inhibition (Mann-Whitney test, *p* value < 0.05) of cell expansion in the dark-grown *sit* mutant compared to the WT (Fig. 4).

### Endogenous ABA levels during germination, skotomorphogenesis and photomorphogenesis

**Fig 5:**
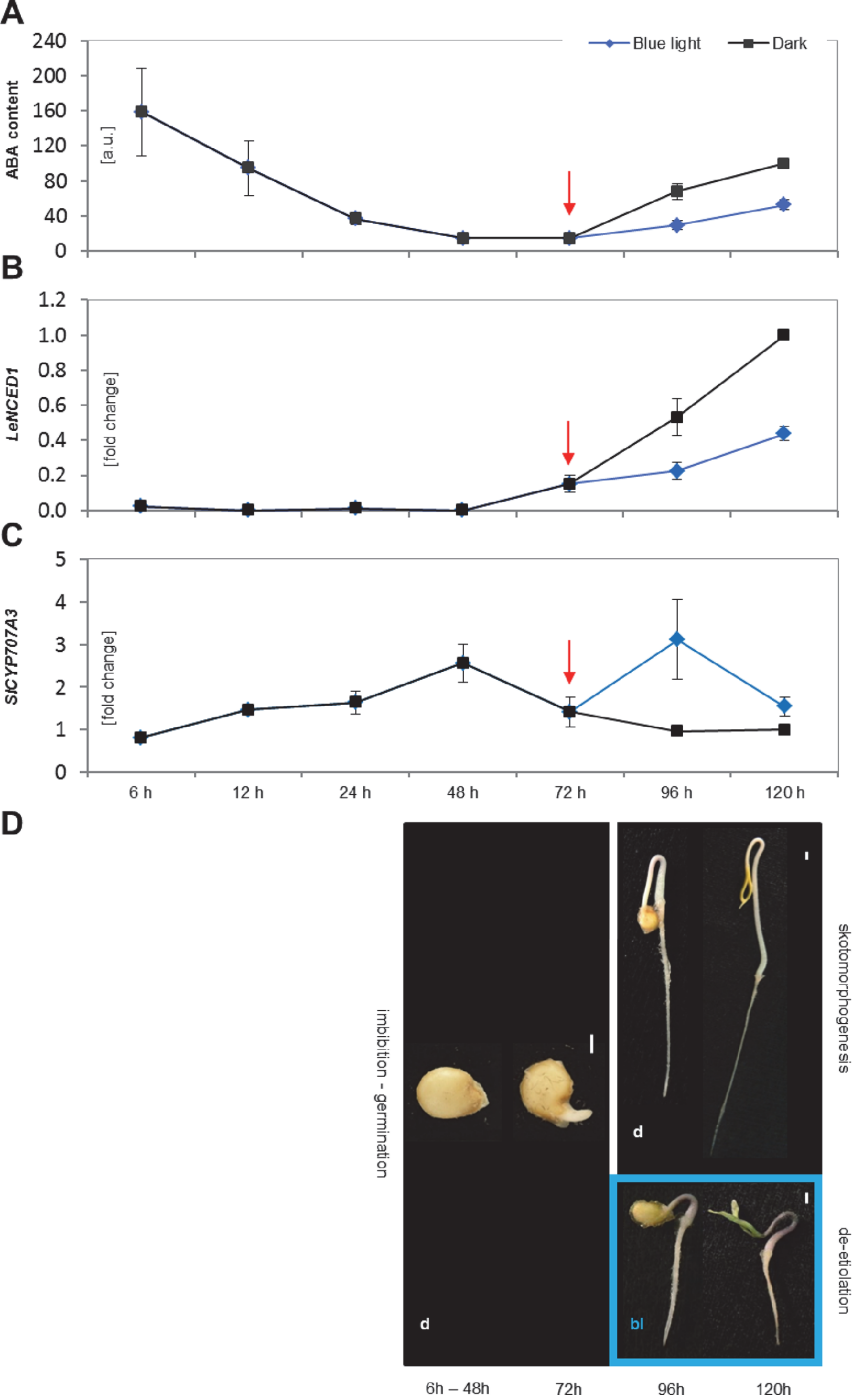
Regulation of ABA metabolism during seed germination and post-germination growth. Free ABA levels are reported as relative means [a.u.] ± SE based on three independent experiments. All values are expressed relative to those for the dark-grown sample at the 120 hours time point, which was assigned a value of 100 a.u. (A). The fold changes in the expression of the *LeNCED1* (B) and *SlCYP707A3* (C) genes are reported as the means of three independent experiments ± SE. *Tip41like* and *PP2ACs* were used as housekeeping genes. Relative quantification was performed using the expression levels for the dark-grown sample at 120 hours as a reference. The red arrow marks the point of seed germination. Figure D) shows the different stages of tomato seedling development considered in this work. The first is seed imbibition (6h–48h), which is followed by seed germination (72h). Both of these steps occurred in darkness in all experiments. The seedlings were then separated (96h–120h) into two groups: one was kept in darkness (d) to induce skotomorphogenesis while the other was grown under continuous BL (bl) to induce de-etiolation. White bars indicate a distance of 1 mm.

The endogenous free ABA contents of whole WT tomato (cv. Rutgers) seedlings were monitored from sowing to early development. The endogenous ABA levels were highest at the first analyzed time point, i.e. 6 hours after sowing, after which they decreased rapidly until germination, 72 hours after sowing. After germination, the ABA content increased gradually in developing seedlings. De-etiolation and photomorphogenesis were initiated by exposure to BL (constant illumination, 10 μmol.m^−2^.s^−1^). Nevertheless, the ABA concentration in dark-grown seedlings was around twice that in seedlings grown under continuous BL and which had undergone de-etiolation and subsequent photomorphogenesis (Fig. 5A).

To determine whether the differences in ABA seen after germination were due to inhibition of ABA biosynthesis or stimulation of ABA degradation, the expression of genes involved in ABA metabolism was investigated. The gene *LeNCED1* encodes 9-cis-epoxycarotenoid dioxygenase, which is the key enzyme involved in ABA synthesis; its expression over time in dark- and BL-grown seedlings is shown in Fig. 5B. The relative abundance of the transcript was below the limit of detection prior to germination but increased progressively in developing seedlings. In addition, the accumulation of *LeNCED1* transcripts in the BL-grown seedlings was almost 60% lower than in those grown in darkness.

We also investigated the expression of four genes which encode the enzymes involved in ABA catabolism (ABA 8´-hydroxylases SlCYP707A1 to SlCYP707A4) [33]. The *SlCYP707A1* transcript was not detected under our conditions, which suggested that the corresponding enzyme was not involved in seedling development. No significant difference was observed between the dark- and BL-grown seedlings with respect to the expression of either *SlCYP707A2* or *SlCYP707A4* (S4 Fig.). But a BL-induced response was observed in *SlCYP707A3* (Fig. 5C). Indeed, this transcript accumulated gradually and at the same rate in the dark- and BL-grown seedlings during the first 48 hours of the experiment (i.e. the seed imbibition period) and then declined between 48 and 72 hours (the point at which germination occurred). In the dark-grown seedlings, the expression of *SlCYP707A3* then declined further before reaching a low basal level. Conversely, in the BL-grown seedlings, its expression increased dramatically between 72 and 96 hours before falling again. The developmental stages of tomato germination and post-germination growth considered in this work are shown in Fig. 5D.

### The effects of ABA on DNA endoreduplication in etiolated tomato hypocotyls

**Fig 6:**
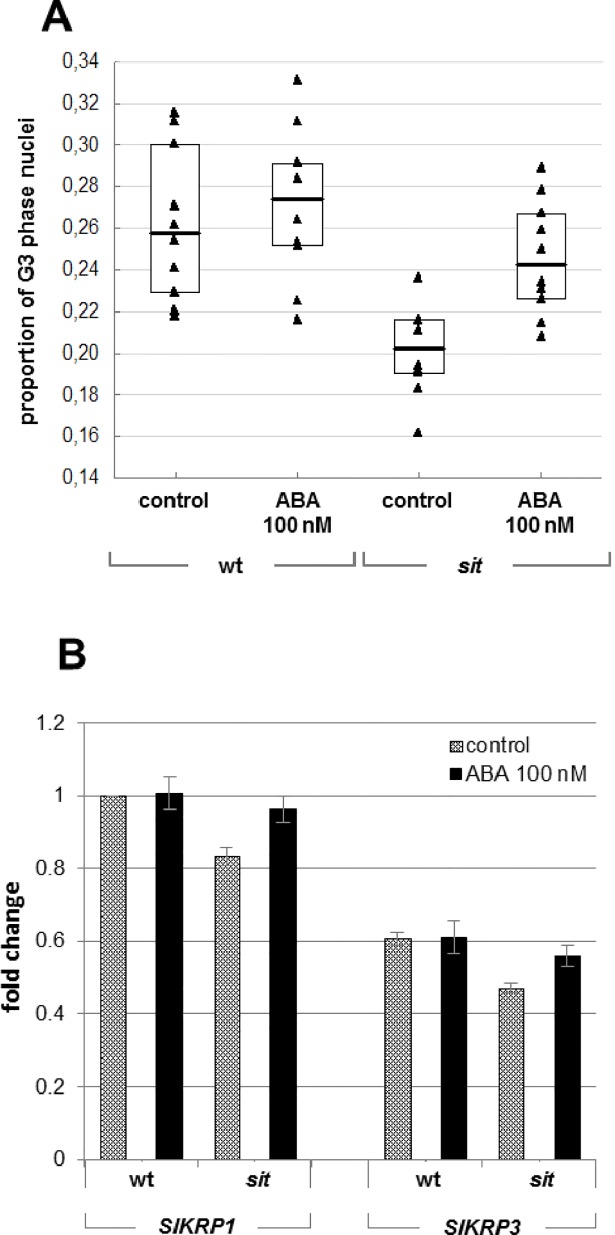
The effects of ABA on endoreduplication. The influence of ABA on nuclear ploidy was investigated using hypocotyl segments of *sit* and WT seedlings grown in darkness on untreated media and media containing 100 nM ABA (A). Triangles represent all measured values (n = 40) for each genotype and treatment. The bars within the boxes indicate the median values in each case, while the boxes’ upper and lower boundaries indicate the boundaries of the first and third quartiles. Three independent experiments were performed. The Kruskal-Wallis ANOVA test with multiple comparisons revealed that only the *sit* control sample differed significantly from the WT control (p < 0.01). No other significant differences were found. (B) Analysis of the expression of the *SlKRP1* and *SlKRP3* genes based on the mean of three independent experiments ± SE. *Tip41like* and *PP2ACs* were used as housekeeping genes. All expression values are quoted relative to those for the “WT control” sample.

The results presented above indicated that ABA plays an important role in regulating dark-induced growth. Etiolated hypocotyl growth is primarily due to cell elongation, with cell division only being important in the development of stomata [34]. Moreover, as in *Arabidopsis*, the tomato hypocotyl elongates along a basipetal gradient. Consequently, the upper region of hypocotyl (i.e. the part situated just above the hook and cotyledons) is the active site of elongation [35]. We therefore investigated the effects of ABA on endoreduplication in this part of the seedlings. For this purpose, 10 mm-long segments were excised from the upper parts of the hypocotyls of the etiolated seedlings of both the *sit* mutant and the corresponding WT, and their cell ploidy was measured by flow cytometry. The cell ploidy status was expressed as the ratio of the number of nuclei in the G3 phase to the total number of nuclei [8C/(2C+4C+8C)], where G3 represents nuclei in the endoreduplication cycle (8C). Our data clearly showed that the extent of endoreduplication in hypocotyl segments from dark-grown untreated *sit* mutants was significantly lower than in the corresponding WT samples. Treatment with exogenous ABA significantly increased the number of G3-phase nuclei in the *sit* hypocotyls but caused only a slight increase in the WT samples (Fig. 6A).

Endoreduplication is controlled by the activity of cyclin-dependent kinase (CDK) proteins, and it has been reported that the expression of the CDK inhibitor *ICK1* is induced by ABA in *Arabidopsis* [36]. Analyses of the expression of two tomato *ICK1* orthologs, *SlKRP1* and *SlKRP3*, [37], [38] indicated that their transcripts were less abundant in the *sit* mutant than in WT plants. Treatment with ABA stimulated the expression of both genes in *sit*, but had no effect on the WT. The overall *SlKRP1* expression was approximately twice that for *SlKRP3* (Fig. 6B).

### The effect of ABA on endogenous cytokinin levels

**Fig 7:**
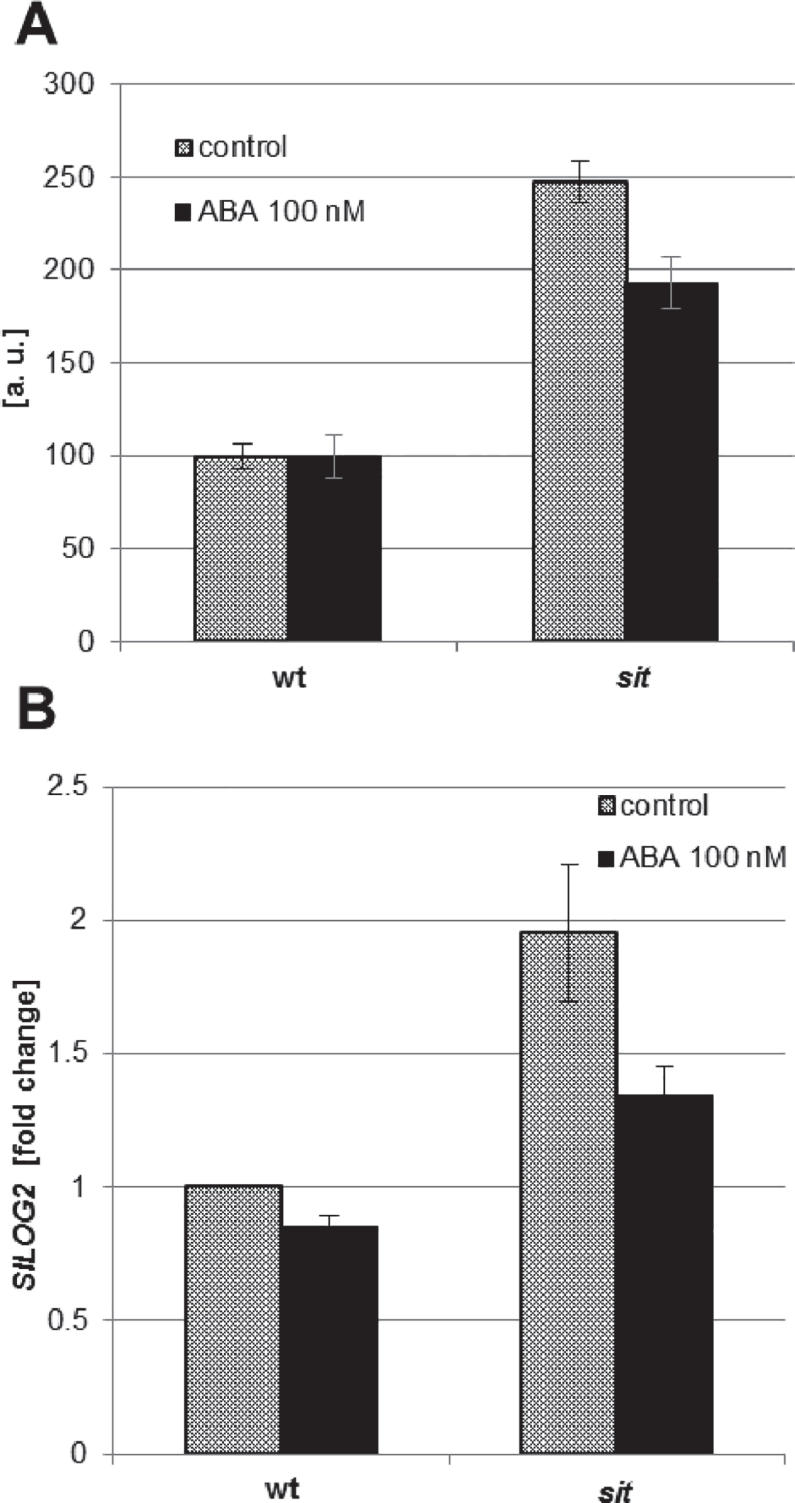
The effects of ABA deficiency on biosynthesis and overall CK levels in etiolated tomato seedlings. Overall CK levels are quoted as the mean of the relative values [a.u.] ± SE for three independent experiments. All measurements are expressed in relation to those for the “WT control” sample, which was assigned a value of 100a.u. (A). The expression of the *SlLOG2* gene is presented as the mean of three independent experiments ± SE. *Tip41like* and *PP2ACs* were used as housekeeping genes. Expression was quantified in relation to that of the specified reference genes in the “WT control” sample (B).

It was recently demonstrated that cytokinins (CKs) accumulate during de-etiolation, probably due to the accumulation of *SlLOG2* transcripts, which encode an enzyme responsible for the synthesis of CK free bases. Moreover, it was evidenced that CK accumulation correlated with the inhibition of endoreduplication [35]. To test the hypothesis that CKs and ABA may act antagonistically during de-etiolation, we measured the endogenous CK contents of dark-grown whole WT and *sit* seedlings grown in the presence and absence of exogenous ABA (Fig. 7A; S4 Table). The *sit* seedlings had much higher levels of CK compounds than the WT plants, and treatment with ABA reduced the abundance of CKs in the mutant but not in the WT. In both cases, the seedlings’ CK levels correlated with the expression of the *SlLOG2* gene, which was expressed in the hypocotyl segments of the *sit* mutant almost twice as strongly as in the WT. Treatment with exogenous ABA significantly inhibited *SlLOG2* transcript accumulation in the *sit* mutant but had only minor effects on the WT (Fig. 7B).

## Discussion

The role of ABA in shoot development has been the subject of debate for some time. While some recent authors still contend that the physiological role of ABA is to inhibit shoot growth [2], [8], [39] there is a growing body of evidence suggesting that it can in fact stimulate shoot growth, or at least contribute to its maintenance [12], [13], [21], [40], [41]. In the present study, the significant inhibition of hypocotyl growth was observed in ABA deficient etiolated seedlings. We found that exogenous ABA stimulated the growth of ABA deficient mutants and had no effect on WTs in nM concentration range. Only a high concentration (5 μM) inhibited WTs growth. As mentioned by Sharp (2002), the interpretation of data related to the application of exogenous ABA are complicated by the uncertain effects of the exogenous treatment [42]. However, our data obtained through treatment with ABA in a wide concentration range suggested that the key factor is plant sensitivity to ABA and homeostatic regulation. It is highly probable that in WT seedlings the number of active ABA receptors corresponds to the signal compound concentration. We can hypothesize that in WT seedlings, the receptors are already saturated by endogenous ABA and the application of exogenous ABA either cannot trigger further response or it can trigger an “over-dose” response that leads to growth inhibition. In ABA deficient seedlings, the endogenous ABA content is too low to activate or saturate the receptors. Thus the *sit* and *not* seedlings are able to respond efficiently to the exogenous ABA treatment. It is important to consider not only the presence or absence, but also the quantity of the signal within the living organism. Then the same compound could evoke opposite responses depending on the concentration used, as has been previously shown for auxin [43] or cytokinins [44]. This can also explain the different sensitivity of *sit* and *not* mutants to ABA. Whereas *sit* seedlings are impaired in the last step of ABA synthesis, the *not* mutation affects the earlier step of conversion of 9´-*cis*-neoxanthin to xanthoxin (LeNCED enzyme). While both mutants produce consistently less ABA than WTs [45], [46], [47], their endogenous ABA content differs (4% and 13% of the WTs for *sit* and *not*, respectively). Thus the difference in sensitivity observed between the two mutants can reflect the difference in their endogenous ABA content. The dose-dependent increase of hypocotyl growth of the mutants suggests that a homeostatic imbalance could be restored by exogenous ABA. Our results suggested that in plants with normal (physiological) ABA levels, additive exogenous ABA will lead to an “over-dose” effect causing growth inhibition, but in the ABA deficient plants the ABA action could be revealed in a more physiological context.

To see the effects of ABA deficiency on the hypocotyl growth the application of ABA biosynthetic inhibitor was performed also as an alternative to genetic approach. Although possible unspecific effects of the chemical treatment should be considered, the application of fluridone led to reduced hypocotyl elongation in dark-grown WT seedlings that further supported the data obtained in genetic experiments. The tomato *sit* mutant was an important genetic tool in this work because it is deficient in the very final step of ABA biosynthesis. Thus, results obtained on this mutant clearly identified the role of ABA in the studied physiological processes. Under conditions with no water stress, the ABA deficient seedlings grown in the dark had significantly shorter hypocotyls than WT plants grown under the same conditions. Previous analysis of the *Arabidopsis aba1* loss-of-function mutant also indicated that ABA may significantly contribute to the process of skotomorphogenesis [16]. However, because the ABA1 enzyme catalyzes an early step in ABA biosynthesis (Fig. 1) whose product is also a precursor of carotenoids, the authors concluded that one of the carotenoid precursors of ABA is required for etiolated growth rather than ABA itself. In the present study, very similar results were obtained with the tomato *sit* mutant, which was deficient in the final step of ABA synthesis, i.e. the conversion of ABA-aldehyde into ABA. Therefore our results strongly suggest that ABA rather than one of its carotenoid precursors promotes the hypocotyl elongation observed during skotomorphogenesis.

Plants defective in ABA signalling are also a good material to investigate the role of ABA during vegetative development. The family of *abscisic-acid insensitive* (*abi*) mutants was the first group of plants discovered with impaired ABA signal transduction [48]. To our knowledge only *abi5* was tested for etiolated hypocotyl elongation and no significant difference was observed between mutant and WT. Even the overexpression of *ABI5* construct in the WT did not change the hypocotyl growth [39]. However, it is hard to determine how the ABA signalling is involved in skotomorphogenesis because of the high level of redundancy between ABA signalling components [49], [50], [51]. In light-grown seedlings a strong inhibition of growth was reported for the *abi8* mutant [52]. Among the ABA receptors the most highly expressed in most developmental stages are PYR1, PYL1, PYL4, PYL8, PYL5, and PYL2. The sextuple mutant showed a strong inhibition of vegetative growth in adult *Arabidopsis* plants [53]. It corresponds to the results obtained with ABA deficient *Arabidopsis* [13] and tomato [12] plants, confirming the importance of proper ABA signalling for maintaining shoot growth.

The postulated correlation between ABA content and tomato hypocotyl growth was further supported by the measurement of endogenous ABA in dark-grown seedlings and in seedlings grown under continuous BL. The dark-grown seedlings undergoing skotomorphogenesis accumulated substantially higher amounts of ABA than those undergoing photomorphogenesis. The fact that seedlings growing under BL still accumulate ABA supports the idea that ABA contributes to the maintenance of steady-state seedling growth. Analyses of the expression of genes involved in ABA synthesis and catabolism suggested that both processes contribute to the regulation of ABA by light. Thus, the transcript of the *LeNCED1* gene, which is involved in ABA synthesis, was undetectable prior to seed germination (in accordance with the fact that ABA in the tomato seeds was synthesized during their maturation) but accumulated during the development of the young seedlings. The accumulation of *LeNCED1* transcripts in the BL-grown seedlings was considerably lower than in the etiolated seedlings. The differential accumulation of *LeNCED1* transcripts observed between dark- and BL-grown seedlings correlated with their differential accumulation of endogenous ABA. The analysis of catabolic genes revealed that *SlCYP707A1* was not detected in our experimental conditions, consistent with its preferential localization in pollinated ovaries [33]. Under our conditions, *SlCYP707A3* was the only ABA 8’-hydroxylase whose expression differed appreciably between dark- and BL-grown tomato hypocotyls, suggesting that this enzyme plays a role during the onset of photomorphogenesis. Continuous exposure to BL led to a decrease of ABA accumulation in the seedlings, driven by the reduction in synthesis and the strong but transient stimulation of catabolism. This suggests that the BL-induced reduction or limitation of endogenous ABA is important in de-etiolation and the onset of photomorphogenesis. Of course, an analysis of protein levels and enzymatic activities would be required to confirm our conclusions. However, we are not the first to report that light regulates endogenous ABA. Indeed, Kraepiel and co-authors demonstrated that the pea phytochrome-deficient *pew-1* mutant had a higher content of ABA in its seeds and leaves than the WT, suggesting that light plays an important role in regulating ABA accumulation [54]. Similarly, Weatherwax and co-authors reported that red light reduced the concentration of ABA in etiolated *Lemna gibba* and also that dark treatment of previously light-grown *Arabidopsis* and *Lemna gibba* plants caused significant ABA accumulation [55]. Moreover, Symons and Reid observed reductions in the ABA content of etiolated pea seedlings after 4 hours of exposure to white light [56].

Skotomorphogenesis is characterized by the rapid elongation of the hypocotyl across a basipetal gradient. This elongation is mainly due to cell expansion, with cell division being restricted to the development of stomata [34]. It has been described previously that leaf epidermal cells of the *sit* mutant are significantly reduced [57]. In the present study, we demonstrated that the shorter hypocotyls of the mutants are due to the reduction of cell expansion. Cell expansion is triggered by two processes: an increase in the cell ploidy by endoreduplication and the expansion of the cell itself, which is driven by water uptake [58]. Whereas there is no doubt about the importance of ABA for water regulation, in this study we focused on the possible influence of ABA on endoreduplication. DNA endoreduplication is a consequence of cell multiplication without division, leading to increased ploidy of the cell. Cell division is regulated via the activity of cyclin-dependent kinases (CDKs) which are regulated by cyclin D-type proteins (CYCD). The initiation of endoreduplication requires the suppression of proteins of the CDK B-family. The activity of the CDK-CYC complexes can be blocked by the inhibitor of CDK/KIP-related (ICK/KRP) proteins. The expression of ICK/KRPs stimulated endoreduplication restricts the G2/M transition [59]. Recently, Bergougnoux and co-authors reported that the switch from skoto- to photomorphogenesis is characterized by the inhibition of endoreduplication [35]. Similarly to studies of ABA effects on shoot growth, existing reports concerning the influence of ABA on endocycles are also contradictory. For example, del Castelano and co-authors reported that treatment with high concentrations (μM) of exogenous ABA inhibited endocycles in *Arabidopsis* leaf primordia, but not in root meristems [60]. Conversely, different studies suggested that ABA promoted endoreduplication by up-regulating the expression of *ICK/KRP*s in *Arabidopsis* [36] and in an alfalfa cell suspension culture [61]. In our study, the ABA deficiency of the *sit* mutant correlated to a lower expression of the *SlKRP1* and *SlKRP3* genes compared to the WT, and to reduced endoreduplication. The expression of *SlKRPs* as well as the level of endoreduplication was decreased by about 20% in *sit* compared to the WT, corresponding to the growth reduction rate observed in this mutant. Such correlation suggests a direct link between endoreduplication and hypocotyl growth. Overall, these results prompted us to hypothesize that the stimulation of endoreduplication is at least one important aspect of the mechanism by which ABA triggers hypocotyl elongation during skotomorphogenesis.

The postulated growth-promoting effect of ABA is supported by the work of Sharp and co-authors [12]. The authors found that under well-watered, non-stressed conditions, the ABA deficient tomato *flacca* mutant exhibited impaired leaf growth and ethylene content two times higher than that of the WT. Exogenous ABA partially restored the mutant’s growth and significantly reduced the accumulation of ethylene. This led to the hypothesis that endogenous ABA maintains shoot elongation in adult tomato plants in a non-hydraulic way by restricting ethylene production. Moreover, studies of the ABA-deficient and ethylene-insensitive *Arabidopsis aba2-1/etr1-1* mutant suggested ethylene-independent mechanisms for the maintenance of shoot growth [13], which implies that some other hormone may be responsible for the relevant processes.

Interaction between ABA and other phytohormones is expected to contribute to the control of stem growth during skotomorphogenesis. Aside from its ethylene-suppressing effects, ABA is also known to be antagonistic to gibberellins (GAs) during seed germination. However, GAs stimulate etiolated hypocotyl growth [62] and are rapidly degraded in response to light [56]. It is more likely that CKs counteract the effects of ABA in stimulating hypocotyl growth, in keeping with their established role during plastid development [63]. Recently it was shown in *Arabidopsis* that CK counteracts ABA by degrading the ABI5 protein [64]. The inhibitory effects of CKs on endoreduplication in tobacco and petunia leaves were reported [65]. Recently the negative regulation of endoreduplication by CKs in the *Arabidopsis* shoot apical meristem has been shown by Scofield and co-authors [66]. This response seems to be shoot-specific since the CK-dependent stimulation of endocycling activity in roots has been reported [67]. Here, we focused on ABA-CK interaction as it was found that CKs inhibit endoreduplication and cell expansion during de-etiolation in tomato seedlings [35]. Indeed the he exposure of etiolated tomato seedlings to BL induced the accumulation of CK in the elongating zone of the hypocotyl and the accumulation of *SlLOG2* transcripts; *SILOG2* encodes an enzyme that is responsible for the synthesis of CK free bases. This is accompanied by the inhibition of endoreduplication, prompting the conclusion that CKs inhibit stem growth by inhibiting endoreduplication and other processes. Our results showed that ABA stimulates endoreduplication and thereby contributes to stem elongation in dark-grown seedlings. This clarifies the previously discussed antagonism between ABA and CKs during seedling development. Indeed, ABA is known to stimulate the accumulation of cytokinin oxidase/dehydrogenase transcripts which encode the proteins responsible for CK degradation [68], while CKs repress the expression of ABA-associated genes [69], [64]. Our data showed that appropriate control of the endogenous ABA balance is necessary to regulate endogenous CK. The insufficient biosynthesis of ABA in the *sit* mutant led to the up-regulation of *SlLOG2* and the concomitant accumulation of endogenous CKs. These effects were partially reversed by treatment with exogenous ABA. Nevertheless, the fact that ABA treatment fully restored the *sit* growth, while the overall amount of CKs was not suppressed to the WT level, raised several questions. These results provided an insight into the interactions between ABA and CKs during skotomorphogenesis, but a more focused study will be required to fully clarify the regulation of CK metabolism.

In conclusion, the results presented here demonstrate that ABA is important for promoting the rapid growth of tomato hypocotyls during skotomorphogenesis. Consistent with previous reports on adult plants we have shown that ABA is necessary to promote growth in young seedlings during the skotomorphogenic stage of development. It seems that this response is masked by homeostasis of endogenous ABA in the WT and can only be observed in ABA deficient mutants. On the other hand, in the WT seedlings the significant increase in endogenous ABA levels could be observed during fast etiolated growth compared to the growth of de-etiolated seedlings. This shows the importance of ABA for cell elongation. Based on our results, we hypothesize that finely-tuned control of ABA metabolism during skotomorphogenesis promotes hypocotyl growth: ABA acts by stimulating the expression of the CDK inhibitors SlKRP1 and SlKRP3 and by inhibiting CK biosynthesis, both of which ultimately stimulate endoreduplication and cell expansion. While the full mechanisms of ABA action during skoto- and photomorphogenesis remain to be determined, our observations shed new light on the role of ABA during the development of young seedlings, and strongly suggest that at least in some stages of tomato seedling development, ABA stimulates growth.

## Supporting Information

S1 Table The hypocotyl length values used for data normalization. (PDF)

S2 Table Absolute quantification of free ABA. (PDF)

S3 Table Absolute quantification of free ABA in hypocotyls of ABA-deficient mutants. (PDF)

S4 Table Absolute quantification of CK levels. (PDF)

S1 File Supporting Information Tables. Table A in S1 File Fold change of *LeNCED1* transcript expression. Table B in S1 File Fold change of *SlCYP707A3* transcript expression. Table C in S1 File Fold change of *SlKRP1* and *SlKRP3* transcript expression. Table D in S1 File Fold change of *SlLOG2* transcript expression.
(PDF)

S1 Fig The hypocotyl elongation of the *not* mutant treated with various concentrations of ABA. (PDF)

S2 Fig The effect of the *sit* mutation on hypocotyl length in BL-grown seedlings. (PDF)

S3 Fig The endogenous ABA content in fluridone treated WT hypocotyls (cv. Rutgers). (PDF)

S4 Fig The effect of fluridone on hypocotyl growth in dark-grown seedlings. (PDF)

S5 Fig The relative expression of the *SlCYP707A2* and *SlCYP707A4* genes. (PDF)

## References

[1] Hansen H, Grossmann K (2000) Auxin-induced ethylene triggers abscisic acid biosynthesis and growth inhibition. Plant Physiol 124: 1437–1448. 11080318 10.1104/pp.124.3.1437 PMC59240

[2] Davies PJ (2010) The plant hormones: their nature, occurrence, and functions In: Davies PJ, editor. Plant hormones: biosynthesis, signal transduction, action! Dordrecht, The Netherlands: Springer. 9–11 p.

[3] Rai MK, Shekhawat NS, Gupta AK, Phulwaria M, Ram K, et al (2011) The role of abscisic acid in plant tissue culture: a review of recent progress. Plant Cell Tiss Org 106: 179–190.

[4] Zhang J, Davies WJ (1990) Does ABA in the xylem control the rate of leaf growth in soil-dried maize and sunflower plants? J Exp Bot 41: 1125–1132.

[5] Creelman RA, Mason HS, Bensen RJ, Boyer JS, Mullet JE (1990) Water deficit and abscisic acid cause differential inhibition of shoot versus root growth in soybean seedlings: Analysis of growth, sugar accumulation, and gene expression. Plant Physiol 92: 205–214. 16667248 10.1104/pp.92.1.205 PMC1062271

[6] Munns R, Cramer GR (1996) Is coordination of leaf and root growth mediated by abscisic acid? Opinion. Plant Soil 185: 33–49.

[7] Wakabayashi K, Sakurai N, Kuraishi S (1989) Role of the outer tissue in abscisic acid‐mediated growth suppression of etiolated squash hypocotyl segments. Physiol Plantarum 75: 151–156.

[8] Hayashi Y, Takahashi K, Inoue S-I, Kinoshita T (2014) Abscisic acid suppresses hypocotyl elongation by dephosphorylating plasma membrane H(+)-ATPase in Arabidopsis thaliana. Plant Cell Physiol 55: 845–853. 10.1093/pcp/pcu028 24492258

[9] Quarrie SA (1987) Use of genotypes differing in endogenous abscisic acid levels in studies of physiology and development In: Hoad GV, Lenton J R, Jackson MB, Atkin RK, editors. Hormone action in plant development—a critical appraisal. London: Butterworths pp. 89–105. 10.1111/tpj.12742

[10] Takahashi K (1972) Abscisic acid as a stimulator for rice mesocotyl growth. Nat New Biol 238: 92–93.

[11] Saab IN, Sharp RE, Pritchard J, Voetberg GS (1990) Increased endogenous abscisic acid maintains primary root growth and inhibits shoot growth of maize seedlings at low water potentials. Plant Physiol 93: 1329–1336. 16667621 10.1104/pp.93.4.1329 PMC1062676

[12] Sharp RE, LeNoble ME, Else MA, Thorne ET, Gherardi F (2000) Endogenous ABA maintains shoot growth in tomato independently of effects on plant water balance: evidence for an interaction with ethylene. J Exp Bot 51: 1575–1584. 11006308 10.1093/jexbot/51.350.1575

[13] LeNoble ME, Spollen WG, Sharp RE (2004) Maintenance of shoot growth by endogenous ABA: genetic assessment of the involvement of ethylene suppression. J Exp Bot 55: 237–245. 14673028 10.1093/jxb/erh031

[14] Barrero JM, Piqueras P, González-Guzmán M, Serrano R, Rodríguez PL, et al (2005) A mutational analysis of the *ABA1* gene of *Arabidopsis thaliana* highlights the involvement of ABA in vegetative development. J Exp Bot 56: 2071–2083. 15983017 10.1093/jxb/eri206

[15] Arsovski AA, Galstyan A, Guseman JM, Nemhauser JL (2012) Photomorphogenesis. In The Arabidopsis Book. e0147. 10.1199/tab.0147 PMC3350170 22582028

[16] Barrero JM, Rodríguez PL, Quesada V, Alabadí D, Blázquez MA, et al (2008) The *ABA1* gene and carotenoid biosynthesis are required for late skotomorphogenic growth in *Arabidopsis thaliana* . Plant Cell Environ 31: 227–234. 17996011 10.1111/j.1365-3040.2007.01759.x

[17] Taylor IB, Linforth RST, Al-Naieb RJ, Bowman WR, Marples BA (1988) The wilty tomato mutants *flacca* and *sitiens* are impaired in the oxidation of ABA-aldehyde to ABA. Plant Cell Environ 11: 739–745.

[18] Tal M, Nevo Y (1973) Abnormal stomatal behavior and root resistance, and hormonal imbalance in three wilty mutants of tomato. Biochem Genet 3: 291–300. 4701995 10.1007/BF00486182

[19] Neill SJ, Horgan R (1985) Abscisic acid production and water relations in wilty tomato mutants subjected to water deficiency. J Exp Bot 36: 1222–1231.

[20] Burbidge A, Grieve TM, Jackson A, Thompson A, McCarty DR, et al (1999) Characterization of the ABA‐deficient tomato mutant notabilis and its relationship with maize Vp14. Plant J 17: 427–431. 10205899 10.1046/j.1365-313x.1999.00386.x

[21] Thompson AJ, Thorne ET, Burbidge A, Jackson AC, Sharp RE, et al (2004) Complementation of notabilis, an abscisic acid-deficient mutant of tomato: Importance of sequence context and utility of partial complementation. Plant Cell Environ 27: 459–471.

[22] Murashige T, Skoog A (1962) A revised medium for rapid growth and bio assays with tobacco tissue cultures. Physiol Plantarum 15: 473–497.

[23] Schneider CA, Rasband WS, Eliceiri KW (2012) NIH Image to ImageJ: 25 years of image analysis. Nat Methods 9: 671–675. 22930834 10.1038/nmeth.2089 PMC5554542

[24] Pěnčík A, Rolčík J, Novák O, Magnus V, Barták P, et al (2009) Isolation of novel indole-3-acetic acid conjugates by immunoaffinity extraction. Talanta 80: 651–655. 10.1016/j.talanta.2009.07.043 19836533

[25] Hoyerová K, Gaudinová A, Malbeck J, Dobrev PI, Kocábek T, et al (2006) Efficiency of different methods of extraction and purification of cytokinins. Phytochem 67: 1151–1159. 16678229 10.1016/j.phytochem.2006.03.010

[26] Dobrev PI, Kamínek M (2002) Fast and efficient separation of cytokinins from auxin and abscisic acid and their purification using mixed-mode solidphase extraction. J Chromatogr A 950: 21–29. 11990994 10.1016/s0021-9673(02)00024-9

[27] Svačinová J, Novák O, Plačková L, Lenobel R, Holík J, et al (2012) A new approach for cytokinin isolation from Arabidopsis tissues using miniaturized purification: pipette tip solid-phase extraction. Plant Methods 8: 17 10.1186/1746-4811-8-17 22594941 PMC3492005

[28] Dekkers BJ, Willems L, Bassel GW, van Bolderen-Veldkamp RP, Ligterink W, et al (2012) Identification of reference genes for RT-qPCR expression analysis in *Arabidopsis* and tomato seeds. Plant Cell Physiol 53: 28–37. 10.1093/pcp/pcr113 21852359

[29] Doležel J, Greilhuber J, Suda J (2007) Estimation of nuclear DNA content in plants using flow cytometry. Nat Protoc 2: 2233–2244. 17853881 10.1038/nprot.2007.310

[30] Parks BM, Cho MH, Spalding EP (1998) Two genetically separable phases of growth inhibition induced by blue light in *Arabidopsis* seedlings. Plant Physiol 118: 609–615. 9765547 10.1104/pp.118.2.609 PMC34837

[31] Parks BM, Spalding EP (1999) Sequential and coordinated action of phytochromes A and B during *Arabidopsis* stem growth revealed by kinetic analysis. P Natl Acad Sci USA 96: 14142–14146. 10570212 10.1073/pnas.96.24.14142 PMC24204

[32] Kitahata N, Asami T (2011) Chemical biology of abscisic acid. J Plant Res 124: 549–557. 10.1007/s10265-011-0415-0 21461661

[33] Nitsch LM, Oplaat C, Feron R, Ma Q, Wolter-Arts M, et al (2009) Abscisic acid levels in tomato ovaries are regulated by *LeNCED1* and *SlCYP707A1* . Planta 299: 1335–1346. 10.1007/s00425-009-0913-7 19322584

[34] Traas J, Hülskamp M, Gendreau E, Höfte H (1998) Endoreduplication and development: rule without dividing? Curr Opin Plant Biol 1: 498–503. 10066638 10.1016/s1369-5266(98)80042-3

[35] Bergougnoux V, Zalabák D, Jandová M, Novák O, Wiese-Klinkenberg A, et al (2012) Effect of blue light on endogenous isopentenyladenine and endoreduplication during photomorphogenesis and de-etiolation of tomato (*Solanum lycopersicum* L.) seedlings. PLoS One. 7, e45255 10.1371/journal.pone.0045255 23049779 PMC3458014

[36] Wang H, Qi Q, Schorr P, Cutler AJ, Crosby WL, et al (1998) ICK1, a cyclin-dependent protein kinase inhibitor from Arabidopsis thaliana interacts with both Cdc2a and CycD3, and its expression is induced by abscisic acid. Plant J 15: 501–510. 9753775 10.1046/j.1365-313x.1998.00231.x

[37] Bisbis B, Delmas F, Joubés J, Sicard A, Hernould M, et al (2006) Cyclin-dependent kinase (CDK) inhibitors regulate the CDK-cyclin complex activities in endoreduplicating cells of developing tomato fruit. J Biol Chem 281: 7374–7383. 16407228 10.1074/jbc.M506587200

[38] Nafati M, Fragne N, Hernould M, Chevalier C, Gévaudant F (2010) Functional characterization of the tomato cyclin-dependent kinase inhibitor SlKRP1 domains involved in protein-protein interactions. New Phytol 188: 136–149. 10.1111/j.1469-8137.2010.03364.x 20618916

[39] Chen H, Zhang J, Neff MM, Hong S-W, Zhang H, et al (2008) Integration of light and abscisic acid signaling during seed germination and early seedling development. P Natl Acad Sci USA 105: 4495–4500 10.1073/pnas.0710778105 18332440 PMC2393781

[40] Mäkelä P, Munns R, Colmer TD, Peltonen‐Sainio P (2003) Growth of tomato and an ABA‐deficient mutant (sitiens) under saline conditions. Physiol Plantarum: 117: 58–63.

[41] Aroca R, Del Mar Alguacil M, Vernieri P, Ruiz-Lozano JM (2008) Plant responses to drought stress and exogenous ABA application are modulated differently by mycorrhization in tomato and an ABA-deficient mutant (*sitiens*). Microb Ecol 56: 704–719. 10.1007/s00248-008-9390-y 18443845

[42] Sharp RE (2002) Interaction with ethylene: changing views on the role of abscisic acid in root and shoot growth responses to water stress. Plant Cell Environ 25: 211–222. 11841664 10.1046/j.1365-3040.2002.00798.x

[43] Taiz L, Zeiger E (2006) Plant Physiology, fourth edition Sunderland, Massachusetts: Sinauer Associates, Inc., Publishers Pp. 485–486. 10.1016/j.ijsu.2006.06.025

[44] Dolezal K, Popa I, Krystof V, Spíchal L, Fojtíková M, et al (2006) Preparation and biological activity of 6-benzylaminopurine derivatives in plants and human cancer cells. Bioorg Med Chem 14: 875–884. 16214355 10.1016/j.bmc.2005.09.004

[45] Jones HG, Sharp CS, Higgs KH (1987). Growth and water relations of wilty mutants of tomato (Lycopersicon esculentum Mill.). J Exp Bot 38: 1848–1856.

[46] Sindhu RK, Walton DC (1988) Xanthoxin metabolism in cell-free preparations from wild type and wilty mutants of tomato. Plant Physiol 88: 178–182. 16666262 10.1104/pp.88.1.178 PMC1055545

[47] Groot SP, Yperen II, Karssen CM (1991) Strongly reduced levels of endogenous abscisic acid in developing seeds of tomato mutant sitiens do not influence in vivo accumulation of dry matter and storage proteins. Physiol Plantarum 81: 73–78.

[48] Leung J, Giraudat J (1998) Abscisic acid signal transduction. Annu Rev Plant Physiol Plant Mol Biol 49: 199–222. 15012233 10.1146/annurev.arplant.49.1.199

[49] Cutler SR, Rodriguez PL, Finkelstein RR, Abrams SR (2010) Abscisic acid: emergence of a core signaling network. Annu Rev Plant Biol 61: 651–679. 10.1146/annurev-arplant-042809-112122 20192755

[50] Rushton DL, Tripathi P, Rabara RC, Lin J, Ringler P, et al (2012) WRKY transcription factors: key components in abscisic acid signalling. Plant Biotechnol J 10: 2–11. 10.1111/j.1467-7652.2011.00634.x 21696534

[51] Finkelstein R (2013) Abscisic acid synthesis and response. Arabidopsis Book 11: e0166 10.1199/tab.0166 24273463 PMC3833200

[52] Brocard-Gifford I, Lynch TJ, Garcia ME, Malhotra B, Finkelstein RR (2004) The Arabidopsis thaliana ABSCISIC ACID-INSENSITIVE8 locus encodes a novel protein mediating abscisic acid and sugar responses essential for growth. Plant Cell 16: 406–421. 14742875 10.1105/tpc.018077 PMC341913

[53] Gonzalez-Guzman M, Pizzio GA, Antoni R, Vera-Sirera F, Merilo E, et al (2012) Arabidopsis PYR/PYL/RCAR receptors play a major role in quantitative regulation of stomatal aperture and transcriptional response to abscisic acid. Plant Cell 24: 2483–2496. 10.1105/tpc.112.098574 22739828 PMC3406898

[54] Kraepiel Y, Rousselin P, Sotta B, Kerhoas L, Einhorn J, et al (1994) Analysis of phytochrome- and ABA-deficient mutants suggests that ABA degradation is controlled by light in Nicotiana plumbaginifolia. Plant J 6: 665–672.

[55] Weatherwax SC, Ong MS, Degenhardt J, Bray EA, Tobin EM (1996) The interaction of light in the regulation of plant gene expression. Plant Physiol 111: 363–370. 8787022 10.1104/pp.111.2.363 PMC157845

[56] Symons GM, Reid JB (2003) Hormone levels and response during de-etiolation in pea. Planta 216: 422–431. 12520333 10.1007/s00425-002-0860-z

[57] Nagel OW, Konings H, Lambers H (1994) Growth rate, plant development and water relations of the ABA deficient tomato mutant *sitiens* . Physiol Plantarum 92: 102–108.

[58] Perrot-Rechenmann C (2010) Cellular responses to auxin: division versus expansion. Cold Spring Harb Perspect Biol. 2: a001446 10.1101/cshperspect.a001446 20452959 PMC2857164

[59] Wen B, Nieuwland J, Murray JAH (2013) The *Arabidopsis* CDK inhibitor ICK3/KRP5 is rate limiting for primary root growth and promotes growth through cell elongation and endoreduplication. J Exp Bot 64: 1–13. 10.1093/jxb/ers358 23440171 PMC3580825

[60] del Castellano M, Boniotti MB, Caro E, Schnittiger A, Gutierrez C (2004) DNA replication licensing affects cell proliferation or endoreplication in a cell type-specific manner. Plant Cell 16: 2380–2393. 15316110 10.1105/tpc.104.022400 PMC520940

[61] Pettko-Szandtner A, Meszaros T, Horvath GV, Bako L, Csordas-Toth E, et al (2006) Activation of an alfalfa cyclin-dependent kinase inhibitor by calmodulin-like domain protein kinase. Plant J 46: 111–123. 16553899 10.1111/j.1365-313X.2006.02677.x

[62] Alabadí D, Gallego-Bartolomé J, García-Cárcel L, Orlando L, Rubio V, et al (2008) Gibberellins modulate light signalling pathways to prevent Arabidopsis seedling de-etiolation in darkness. Plant J 53: 324–335. 18053005 10.1111/j.1365-313X.2007.03346.x

[63] Yamburenko MV, Zubo YO, Vankova R, Kusnetsov VV, Kulaeva ON, et al (2013) Abscisic acid represses the transcription of chloroplast genes. J Exp Bot 64: 4491–4502. 10.1093/jxb/ert258 24078671 PMC3808324

[64] Guan C, Wang X, Feng J, Hong S, Liang Y, et al (2014) Cytokinin antagonizes abscisic acid-mediated inhibition of cotyledon greening of promoting the degradation of ABSCISIC ACID INSENSITIVE5 protein in Arabidopsis. Plant Physiol 164: 1515–1526. 10.1104/pp.113.234740 24443524 PMC3938637

[65] Valente P, Tao W, Verbelen JP (1998). Auxins and cytokinins control DNA endoreduplication and deduplication in single cells of tobacco. Plant Sci 134: 207–215.

[66] Scofield S, Dewitte W, Nieuwland J, Murray JA (2013) The Arabidopsis homeobox gene SHOOT MERISTEMLESS has cellular and meristem‐organisational roles with differential requirements for cytokinin and CYCD3 activity. Plant J 75: 53–66. 10.1111/tpj.12198 23573875

[67] Takahashi N, Kajihara T, Okamura C, Kim Y, Katagiri Y, et al (2013) Cytokinins Control Endocycle Onset by Promoting the Expression of an APC/C Activator in Arabidopsis Roots. Curr Biol 23: 1812–1817. 10.1016/j.cub.2013.07.051 24035544

[68] Brugiere N, Jiao S, Hantke S, Zinselmeier C, Roessler JA, et al (2003) Cytokinin oxidase gene expression in maize is localized to the vasculature, and is induced by cytokinins, abscisic acid, and abiotic stress. Plant Physiol 132: 1228–1240. 12857805 10.1104/pp.102.017707 PMC167063

[69] Rivero RM, Gimeno J, Van Deynze A, Harkamal W, Blumwald E (2010) Enhanced cytokinin synthesis in tobacco plants expressing PSARK:: IPT prevents the degradation of photosynthetic protein complexes during drought. Plant Cell Physiol 51: 1929–1941. 10.1093/pcp/pcq143 20871100

[70] Nambara E, Marion-Poll A (2005) Abscisic acid biosynthesis and catabolism. Annu Rev Plant Biol 56: 165–185.oamdg. 15862093 10.1146/annurev.arplant.56.032604.144046

