# Supplementary material for: Endogenous Abscisic Acid Promotes Hypocotyl Growth and Affects Endoreduplication during Dark-Induced Growth in Tomato (Solanum lycopersicum L.)
Source: PLoS One. 2015 Feb 19;10(2):e0117793. doi: 10.1371/journal.pone.0117793 (PMC4334974; doi:10.1371/journal.pone.0117793)
Supplement: S1 Table — (PDF) [file pone.0117793.s001.pdf]

Supporting table S1 The values of hypocotyl length used for data normalization. Medians, first and third quartiles are given. The control sample used for data normalization is marked as “norm.”

Figure	Genotype and/or treatment	Median hypocotyl length [mm]	1st quartile [mm]	3rd quartile [mm]	
**2A**	WT control	98	91	104	norm.
	WT 100 nM ABA	101	98	103	
	sit control	80	72	84	
	sit 100 nM ABA	99	94	106	
**2B**	control	53	46	62	norm.
	50 nM	76	67	81	
	100 nM	83	78	87	
	500 nM	74	69	80	
	1 µM	74	64	80	
	5 µM	46	38	52	
**2C**	control	73	66	83	norm.
	50 nM	77	67	81	
	100 nM	80	71	92	
	500 nM	74	63	85	
	1 µM	69	56	78	
	5 µM	41	22	58	
**2D**	WT control	101	95	104	norm.
	WT 50 nM ABA	100	91	106	
	not control	91	86	96	
	not 50 nM ABA	103	99	112	
**S1**	control	52	46	64	norm.
	50 nM	81	75	81	
	100 nM	84	77	86	
	500 nM	71	67	76	
	1 µM	75	67	80	
	5 µM	40	36	45	
**S2**	WT	28	22	33	norm.
	sit	26	23	27	
**S4**	control	68	62	73	norm.
	fluridone 10 µM	53	51	57	
