# Supplementary material for: Endogenous Abscisic Acid Promotes Hypocotyl Growth and Affects Endoreduplication during Dark-Induced Growth in Tomato (Solanum lycopersicum L.)
Source: PLoS One. 2015 Feb 19;10(2):e0117793. doi: 10.1371/journal.pone.0117793 (PMC4334974; doi:10.1371/journal.pone.0117793)
Supplement: S2 Table — (PDF) [file pone.0117793.s002.pdf]

Supporting table S2 Absolute quantification of free ABA in tomato seedlings based on three independent experiments ± SE from three technical replicates.

Sample	**Set I.** **ABA** [pmol/g FW]	**Set II.** **ABA** [pmol/g FW]	**Set III.** **ABA** [pmol/g FW]
**6 h – D**	**29** ± 2.5	**9** ± 0.7	**45** ± 3.6
**12 h – D**	**18** ± 5.4	**6** ± 0.4	**25** ± 4.2
**24 h – D**	**5** ± 0.1	**4** ± 0.3	**n. a.**
**48 h – D**	**2** ± 0.2	**2** ± 0.3	**3** ± 0.3
**72 h – D**	**1** ± 0.2	**1** ± 0.1	**7** ± 1.4
**96 h – D**	**8** ± 1.7	**7** ± 0.9	**26** ± 1.0
**96 h – BL**	**5** ± 0.7	**3** ± 0.5	**8** ± 1.2
**120 h – D**	**11** ± 0.5	**15** ± 0.6	**32** ± 0.8
**120 h – BL**	**7** ± 0.2	**8** ± 0.6	**12** ± 1.6

n. a.: not analyzed - sample lost during the analysis
