# Supplementary material for: Endogenous Abscisic Acid Promotes Hypocotyl Growth and Affects Endoreduplication during Dark-Induced Growth in Tomato (Solanum lycopersicum L.)
Source: PLoS One. 2015 Feb 19;10(2):e0117793. doi: 10.1371/journal.pone.0117793 (PMC4334974; doi:10.1371/journal.pone.0117793)
Supplement: S3 Table — (PDF) [file pone.0117793.s003.pdf]

Supporting table S3 Absolute quantification of free ABA in hypocotyls of ABA-deficient mutants and corresponding WTs based on two biological repeats ± SE from two technical replicates.

Sample	**Set I.** ABA [pmol/g FW]	**Set II.** ABA [pmol/g FW]
sit	**0.8** ± 0.2	**0.8**±0.1
**WT** (cv. Rheinlands-Ruhm)	**17.9** ± 0.4	**18** ± 1.9
not	**2.6** ± 0.5	**2.9** ± 0.0
**WT** (cv. Lukullus)	**21.8** ± 0.6	**19.5** ± 1.4
