# Supplementary material for: Endogenous Abscisic Acid Promotes Hypocotyl Growth and Affects Endoreduplication during Dark-Induced Growth in Tomato (Solanum lycopersicum L.)
Source: PLoS One. 2015 Feb 19;10(2):e0117793. doi: 10.1371/journal.pone.0117793 (PMC4334974; doi:10.1371/journal.pone.0117793)
Supplement: S4 Table — (PDF) [file pone.0117793.s004.pdf]

Supporting table S4 Absolute quantification of CK levels in tomato seedlings ± SE based on three independent experiments. <LOD indicates that the levels of the corresponding compound were below the limit of detection; cZ *cis*-zeatin compounds, tZ *trans*-zeatin compounds, DHZ dihydrozeatin compounds, iP isopentenyladenine compounds, xR ribosides, xOG O-glucosides, x9G 9-glucosides, xMP monophosphates, x7G 7-glucosides.

	**wt control**	**wt 100 nM ABA**	***sit* control**	***sit* ABA 100 nM**
**CKs**	[pmol/g FW] ± SE	[pmol/g FW] ± SE	[pmol/g FW] ± SE	[pmol/g FW] ± SE
**cZ**	**0.09** ± 0.03	**0.13** ± 0.01	**0.14** ± 0.02	**0.10** ± 0.01
**cZR**	**0.37** ± 0.04	**0.42** ± 0.05	**1.13** ± 0.40	**0.33** ± 0.05
**cZOG**	**0.09** ± 0.03	**0.13** ± 0.02	**0.14** ± 0.01	**0.08** ± 0.01
**cZ9G**	**0.15** ± 0.00	**0.14** ± 0.01	**0.11** ± 0.02	**0.10** ± 0.01
**cZROG**	**0.15** ± 0.01	**0.15** ± 0.02	**0.41** ± 0.15	**0.18** ± 0.04
**cZRMP**	**0.36** ± 0.01	**0.55** ± 0.20	**0.43** ± 0.14	**0.64** ± 0.23
**cZ7G**	**8.55** ± 0.59	**8.79** ± 0.26	**10.76** ± 0.35	**8.29** ± 0.65
**tZ**	**0.17** ± 0.02	**0.09** ± 0.02	**0.21** ± 0.04	**0.16** ± 0.01
**tZR**	**1.35** ± 0.09	**0.55** ± 0.02	**1.12** ± 0.17	**0.60** ± 0.08
**tZOG**	**0.10** ± 0.02	**0.07** ± 0.02	**0.13** ± 0.03	**0.08** ± 0.01
**tZ9G**	**0.28** ± 0.03	**0.18** ± 0.00	**0.46** ± 0.06	**0.28** ± 0.05
**tZROG**	**0.57** ± 0.06	**0.51** ± 0.09	**1.34** ± 0.07	**1.01** ± 0.10
**tZRMP**	**0.66** ± 0.08	**0.28** ± 0.02	**0.53** ± 0.06	**0.57** ± 0.05
**tZ7G**	**8.26** ± 0.47	**5.33** ± 0.43	**15.44** ± 0.50	**9.34** ± 1.88
**DHZ**	**0.01** ± 0.00	**0.01** ± 0.00	**0.02** ± 0.00	**0.01** ± 0.00
**DHZR**	**0.10** ± 0.01	**0.05** ± 0.01	**0.14** ± 0.02	**0.09** ± 0.05
**DHZOG**	**0.06** ± 0.00	**0.08** ± 0.01	**0.11** ± 0.03	**0.07** ± 0.02
**DHZ9G**	<LOD	<LOD	<LOD	<LOD
**DHZROG**	**0.73** ± 0.08	**1.10** ± 0.38	**2.19** ± 0.17	**1.82** ± 0.50
**DHZRMP**	<LOD	<LOD	<LOD	<LOD
**DHZ7G**	**23.29** ± 1.40	**26.91** ± 4.26	**73.61** ± 0.61	**44.07** ± 3.54
**iP**	**0.24** ± 0.02	**0.42** ± 0.09	**0.33** ± 0.06	**0.32** ± 0.12
**iPR**	**0.12** ± 0.03	**0.14** ± 0.04	**0.31** ± 0.07	**0.26** ± 0.06
**iP9G**	**0.12** ± 0.02	**0.15** ± 0.04	**0.13** ± 0.01	**0.15** ± 0.02
**iPRMP**	**0.40** ± 0.07	**0.31** ± 0.09	**0.49** ± 0.06	**0.41** ± 0.06
**iP7G**	**14.43** ± 0.94	**14.09** ± 1.02	**40.38** ± 3.59	**48.05** ± 1.12
**SUMA**	**60.64** ± 4.02	**60.58** ± 7.12	**150.07** ± 6.63	**117.00** ± 8.66
