# Supplementary material for: Endogenous Abscisic Acid Promotes Hypocotyl Growth and Affects Endoreduplication during Dark-Induced Growth in Tomato (Solanum lycopersicum L.)
Source: PLoS One. 2015 Feb 19;10(2):e0117793. doi: 10.1371/journal.pone.0117793 (PMC4334974; doi:10.1371/journal.pone.0117793)
Supplement: S1 File — Table A in S1 File Fold change of LeNCED1 transcript expression. Table B in S1 File Fold change of SlCYP707A3 transcript expression. Table C in S1 File Fold change of SlKRP1 and SlKRP3 transcript expression. Table D in S1 File Fold change of SlLOG2 transcript expression. (PDF) [file pone.0117793.s005.pdf]

Supporting table A Fold change in *LeNCED1* transcript expression based on three independent experiments.

Sample	Set I. Relative expression	Set II. Relative expression	Set III. Relative expression
**6 h – D**	**0.07**	**0.00**	**0.00**
**12 h – D**	**0.00**	**0.00**	**0.00**
**24 h – D**	**0.04**	**0.00**	**0.00**
**48 h – D**	**0.00**	**0.00**	**0.00**
**72 h – D**	**0.24**	**0.04**	**0.17**
**96 h – D**	**0.75**	**0.30**	**0.54**
**96 h – BL**	**0.32**	**0.12**	**0.23**
**120 h – D**	**1.00**	**1.00**	**1.00**
**120 h – BL**	**0.46**	**0.34**	**0.51**

Supporting Table B Fold change in *SlCYP707A3* transcript expression based on three independent experiments.

Sample	Set I. Relative expression	Set II. Relative expression	Set III. Relative expression
**6 h – D**	**0.60**	**1.13**	**0.72**
**12 h – D**	**1.33**	**1.36**	**1.72**
**24 h – D**	**1.42**	**2.29**	**1.22**
**48 h – D**	**3.64**	**1.90**	**2.14**
**72 h – D**	**1.33**	**2.21**	**0.73**
**96 h – D**	**0.91**	**1.17**	**0.82**
**96 h – BL**	**1.63**	**5.37**	**2.34**
**120 h – D**	**1.00**	**1.00**	**1.00**
**120 h – BL**	**1.73**	**1.02**	**1.88**

Supporting table C Fold change in *SlKRP1* and *SlKRP3* transcript expression based on three independent experiments.

Gene	Sample	Set I. Relative expression	Set II. Relative expression	Set III. Relative expression
SlKRP1	**wt control**	1.00	1.00	1.00
	**wt ABA 100 nM**	0.91	1.00	1.11
	***sit* control**	0.83	0.78	0.89
	***sit* ABA 100 nM**	0.95	0.89	1.04
SlKRP3	**wt control**	0.57	0.60	0.64
	**wt ABA 100 nM**	0.50	0.66	0.67
	***sit* control**	0.46	0.45	0.50
	***sit* ABA 100 nM**	0.55	0.50	0.62

Supporting table D Fold change in *SlLOG2* transcript expression based on three independent experiments.

Sample	**Set I.** **Relative expression**	**Set II.** **Relative expression**	**Set III.** **Relative expression**
**wt control**	1.00	1.00	1.00
**wt ABA 100 nM**	0.76	0.93	0.88
***sit* control**	1.39	2.49	1.98
***sit* ABA 100 nM**	1.12	1.57	1.35
