# Supplementary material for: Endogenous Abscisic Acid Promotes Hypocotyl Growth and Affects Endoreduplication during Dark-Induced Growth in Tomato (Solanum lycopersicum L.)
Source: PLoS One. 2015 Feb 19;10(2):e0117793. doi: 10.1371/journal.pone.0117793 (PMC4334974; doi:10.1371/journal.pone.0117793)
Supplement: S1 Fig — (PDF) [file pone.0117793.s006.pdf]

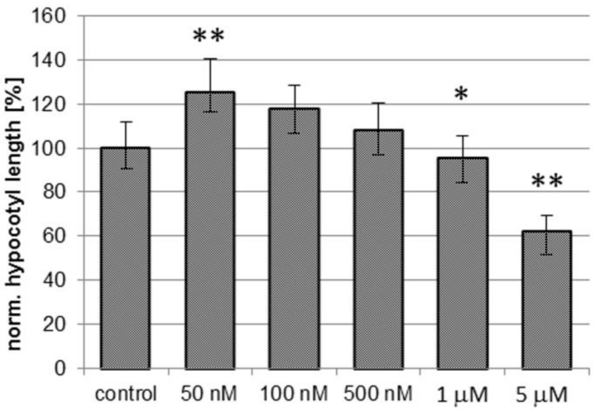

Supporting figure S1 The hypocotyl elongation of *not* mutant treated with various concentrations of ABA. Germinated seeds were transferred on basal media (control) or media supplemented with ABA and grown in the dark for 4 days. The results shown in the figure represent the medians of normalized length of hypocotyls from 1 independent experiment; the error bars represent the boundaries of the first and third quartiles. The sample “control” was set as 100% hypocotyl length and all other values (medians, quartiles) are expressed as percentage of this value. To prove significance the Kruskal-Wallis ANOVA with multiple post-hoc comparison was performed. Asterisks denote values that differ significantly from “control” sample (** $p < 0.01$, * $p < 0.05$; n=135).
