# Supplementary material for: Endogenous Abscisic Acid Promotes Hypocotyl Growth and Affects Endoreduplication during Dark-Induced Growth in Tomato (Solanum lycopersicum L.)
Source: PLoS One. 2015 Feb 19;10(2):e0117793. doi: 10.1371/journal.pone.0117793 (PMC4334974; doi:10.1371/journal.pone.0117793)
Supplement: S2 Fig — (PDF) [file pone.0117793.s007.pdf]

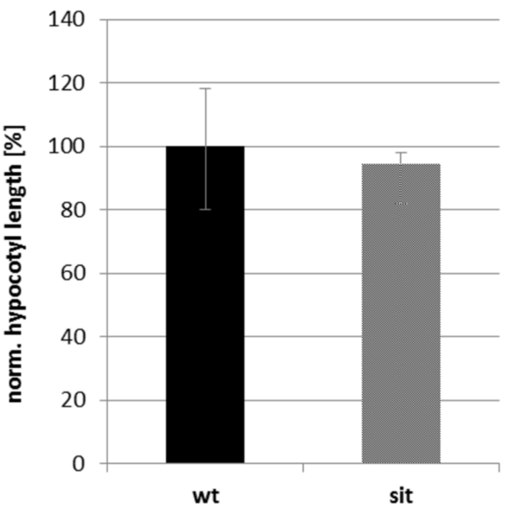

Supporting figure S2 The effect of the *sit* mutation on hypocotyl length in BL-grown seedlings. Seeds of the WT (cv. Rheinlands Ruhm) and *sit* mutant were germinated in darkness and then grown under continuous BL for 4 days. The results shown in the figure represent the medians of normalized length of hypocotyls from 1 independent experiment; the error bars represent the boundaries of the first and third quartiles. The sample "wt" was set as 100% hypocotyl length and all other values (medians, quartiles) are expressed as percentage of this value (Mann-Whitney test, $p > 0.05$, $n=40$).
