# Supplementary material for: Endogenous Abscisic Acid Promotes Hypocotyl Growth and Affects Endoreduplication during Dark-Induced Growth in Tomato (Solanum lycopersicum L.)
Source: PLoS One. 2015 Feb 19;10(2):e0117793. doi: 10.1371/journal.pone.0117793 (PMC4334974; doi:10.1371/journal.pone.0117793)
Supplement: S3 Fig — (PDF) [file pone.0117793.s008.pdf]

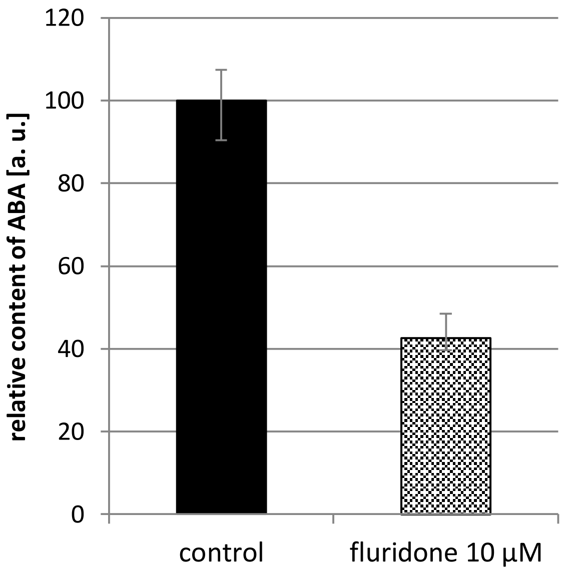

Supporting figure S3 The endogenous ABA content in fluridone treated WT hypocotyls (cv. Rutgers). Free ABA levels are reported as relative means [a.u.] ± SE based on two independent experiments. The control sample was assigned a value of 100 a.u.
