# Supplementary material for: Endogenous Abscisic Acid Promotes Hypocotyl Growth and Affects Endoreduplication during Dark-Induced Growth in Tomato (Solanum lycopersicum L.)
Source: PLoS One. 2015 Feb 19;10(2):e0117793. doi: 10.1371/journal.pone.0117793 (PMC4334974; doi:10.1371/journal.pone.0117793)
Supplement: S4 Fig — (PDF) [file pone.0117793.s009.pdf]

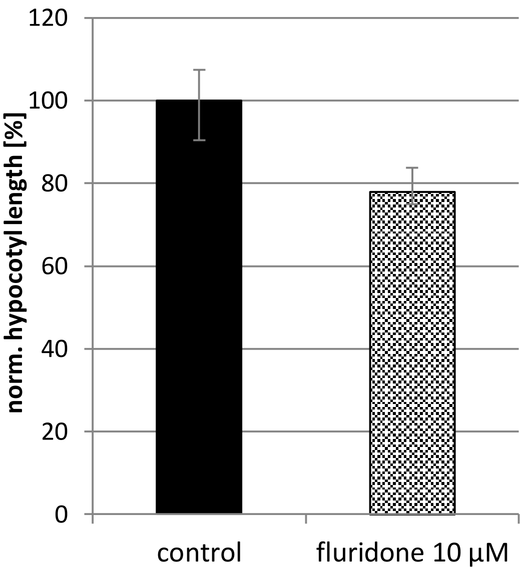


**Supporting figure S4** The effect of fluridone on hypocotyl growth in dark-grown seedlings. Germinated seeds of WT (cv. Rutgers) were transferred to media supplemented with 10 µM fluridone and grown for 4 days in darkness. The results shown in the figure represent the medians of normalized length of hypocotyls from 2 independent experiments; the error bars represent the boundaries of the first and third quartiles. The control sample was set as 100% hypocotyl length. Other values (medians, quartiles) are expressed as percentage of these values. The statistical significance was evaluated by Mann-Whitney U test; $p < 0.02$.
