# Supplementary material for: Endogenous Abscisic Acid Promotes Hypocotyl Growth and Affects Endoreduplication during Dark-Induced Growth in Tomato (Solanum lycopersicum L.)
Source: PLoS One. 2015 Feb 19;10(2):e0117793. doi: 10.1371/journal.pone.0117793 (PMC4334974; doi:10.1371/journal.pone.0117793)
Supplement: S5 Fig — (PDF) [file pone.0117793.s010.pdf]

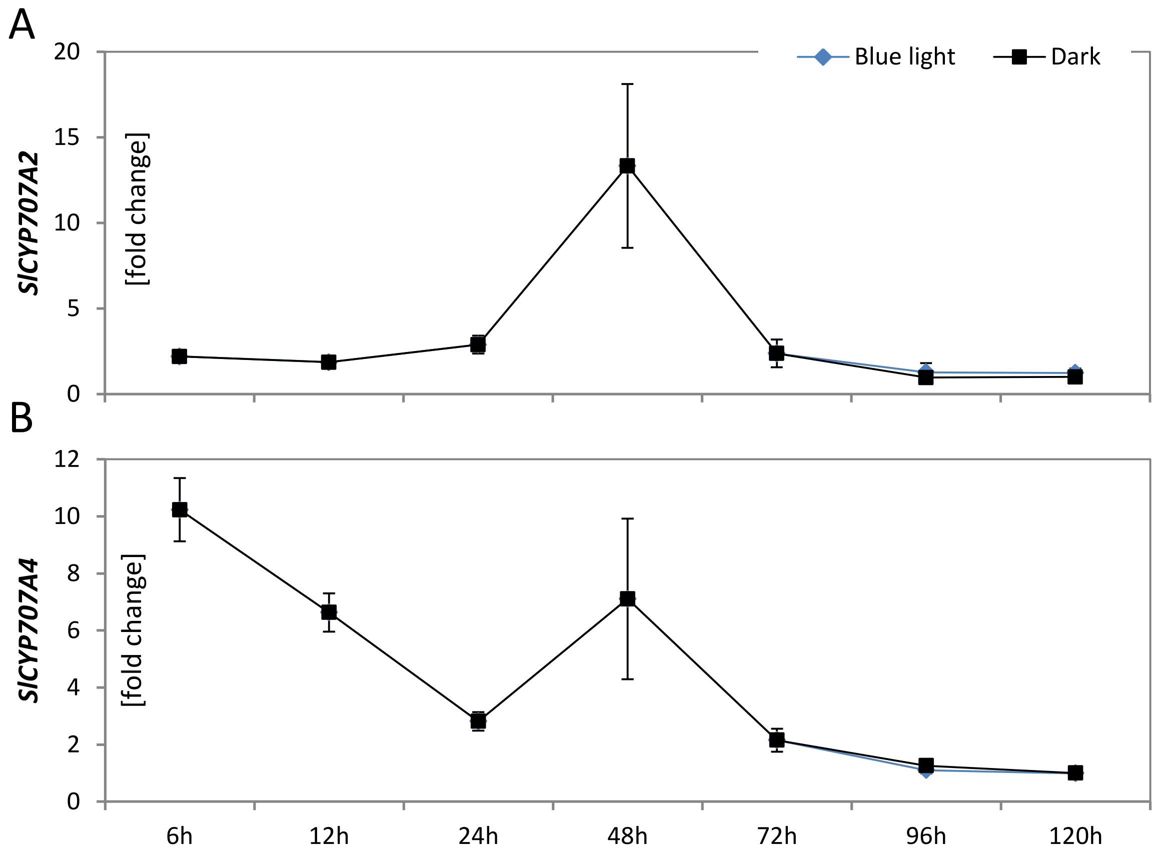


**Supporting figure S5** The relative expression of the A) *SlCYP707A2* and B) *SlCYP707A4* ABA catabolic genes. Values shown are the geometric means of three biological replicates ± SE. *Tip41like* and *PP2ACs* were used as housekeeping genes. All values are expressed in arbitrary units relative to the expression of the corresponding gene in the 120h-Dark” sample.
